# Synthesis of oligo-α-(1→2)-4,6-dideoxy-4-formamido-d-mannopyranosides related to the A epitope of the *Brucella* O-polysaccharide and their use for assaying of serum immunoglobulins

**DOI:** 10.3389/fchem.2025.1662885

**Published:** 2025-08-29

**Authors:** Timur M. Volkov, Yury E. Tsvetkov, Dmitry V. Yashunsky, Anton N. Kuznetsov, Oleg D. Sclyarov, Olesia V. Babicheva, Dmitry O. Zherdev, Liliya I. Mukhametova, Sergei A. Eremin, Vadim B. Krylov, Nikolay E. Nifantiev

**Affiliations:** ^1^ Laboratory of Glycoconjugate Chemistry, N. D. Zelinsky Institute of Organic Chemistry, Russian Academy of Sciences, Moscow, Russia; ^2^ Russian State Centre of Quality and Standardization of Veterinary Drugs and Feeds, Moscow, Russia; ^3^ Department of Chemistry, Moscow State University, Moscow, Russia

**Keywords:** *Brucella*, O-antigen, N-formyl-D-perosamine, antibodies detection, fluorescence polarization assay

## Abstract

Pathogenic bacteria of the genus *Brucella* cause a severe threat for public health and agricultural economics. The World Health Organization considers brucellosis to be one of the most serious and also neglected zoonotic diseases. The use of traditional whole-cell brucellosis vaccines complicates the differentiation between infected and vaccinated animals (DIVA). Moreover, diagnostics based on lipopolysaccharide of *Brucella* are susceptible to false positive results. Structural features of *Brucella* O-antigens make synthetic oligosaccharides promising agents for the development of diagnostic tools and vaccines against brucellosis. Here we report the synthesis of spacer-armed di-, tri-, tetra- and penta-4,6-dideoxy-4-formamido-α-(1→2)-d-mannopyranosides which are related to the A-epitope of *Brucella* O-antigen. The key α-(1→2)-linked disaccharide thioglycoside donor was synthesized by employing the strategy of orthogonal glycosylation of thioglycoside acceptor with trichloroacetimidate donor. Sequential block-wise assembly yielded a series of desired compounds, which were subsequently deprotected and converted into target molecules and then into their fluorescein-labeled conjugates. The obtained conjugates were employed as tracers in a fluorescence polarization assay (FPA) to detect anti-*Brucella* immunoglobulins. Among the studied compounds, the trisaccharide conjugate showed the greatest difference in median FP signals between *Brucella*-positive and *Brucella*-negative sera samples making it a promising candidate for developing FP diagnostic assays. The decreased FP signal in the cases of tetra- and pentasaccharide tracers can be associated with the known “propeller-effect” due to the rotational mobility of the part bearing the fluorescent label and of the fluorescein itself and/or the enlarging of the distance between the fluorescein part and the antibody-oligosaccharide complex. This observation demonstrates the advantages of using synthetic relatively small synthetic tracers with well-defined structure in comparison with heterogeneous fluorescein-labelled O-polysaccharides which are in use today in spite of the fact that they contain poorly characterized amounts of label attached along the polysaccharide chains.

## Introduction

Brucellosis is a zoonotic infection primarily affecting domestic animals, caused by Gram-negative *Brucella spp.* (Khurana et al., 2021). Upon contact with infected animals or their products, the infection can be transmitted to humans, leading to serious acute and chronic illnesses with non-specific symptoms similar to those of malaria or influenza (Franco et al., 2007).

Early diagnosis is crucial for the prevention, control of the spread, and treatment of brucellosis. Currently, its detection is based on several principles (El Ayoubi et al., 2024; Kuznetsov et al., 2025; Tian et al., 2020), including the monitoring of specific antibodies to the *Brucella* O-polysaccharide in animal and human sera. For this purpose the preparations containing *Brucella* O-polysaccharide are used most often as marker antigens in the serodiagnosis of brucellosis (Godfroid et al., 2010). In particular, the systematic review and meta-analysis of the accuracy of serological tests for bovine brucellosis showed that indirect enzyme-linked immunosorbent assay (ELISA) and FPA are relatively easy to perform and interpret, and the test that showed the best overall accuracy was FPA (Andrade et al., 2024). Fluorescence polarization assay (FPA) is a homogeneous technique that involves only a single mix-and-measure step. Results can be obtained within minutes, making FPA a promising method for rapid detection of biomarkers in disease diagnostics (Jolley and Nasir, 2003). The availability of portable instruments capable of measuring fluorescence polarization signals outside the laboratory has made FPA another option for on-site infection detection. FPA has proven to be effective for the detection of antibodies against bacterial contamination, especially for the detection of antibodies to gram-negative bacteria (Jolley and Nasir, 2003).

**FIGURE 1:**
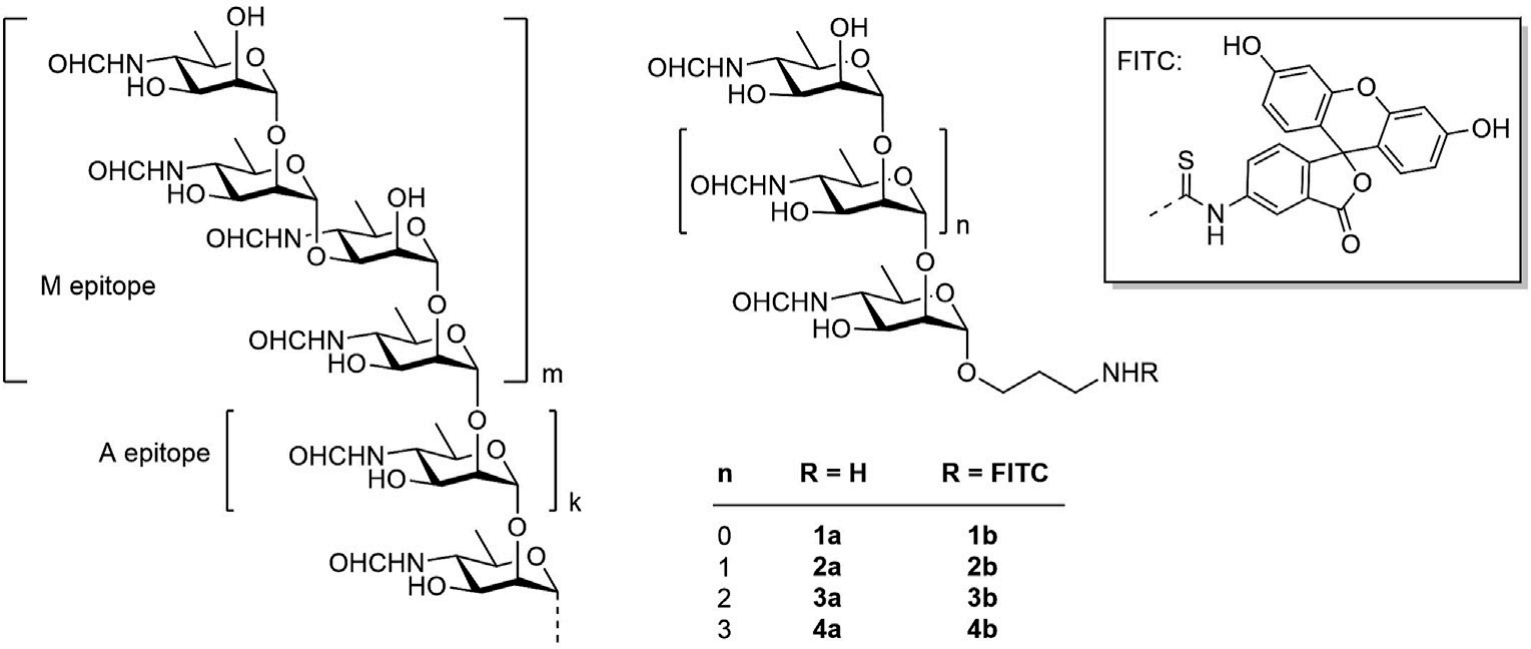
Structure of the *Brucella* O-polysaccharide (on the left), target spacered oligosaccharides **1a**-**4a** and fluorescein labeled conjugates **1b**-**4b** prepared in this work.

The O-polysaccharide is an α-(1→2)-linked polymer of 4,6-dideoxy-4-formamido-d-mannopyranose (perosamine) terminated by one or more tetrasaccharides containing a central α-(1→3)-glycosidic bond (Figure 1, on the left) (Kubler-Kielb and Vinogradov, 2013). The antigenic determinants, represented by the α-(1→2)-linked chain and the terminal tetrasaccharide with the central α-(1→3)-bond, are referred to as the A epitope and the M epitope, respectively (Kubler-Kielb and Vinogradov, 2013). The ratio of A and M epitopic fragments in *Brucella* O-polysaccharides depends on the type of the serotype, and in some cases the M epitope can be even absent (Kubler-Kielb and Vinogradov, 2013).

The use of the natural *Brucella* O-polysaccharide as the diagnostic antigen has some disadvantages. In particular, the cultivation of bacteria and the isolation, purification, and standardization of lipopolysaccharides is a challenging task. Another complication is related to the existence of bacterial species that produce O-polysaccharides made from N-acylated d-perosamine. These include, for example, *Yersinia enterocolitica* O:9 (Caroff et al., 1984), *Escherichia coli* 0157 (Perry and Bundle, 1990), *Vibrio cholerae* O1 (Kenne et al., 1982), and some others. Infection of animals and humans by these bacteria induces the production of antibodies that can cross-react with the *Brucella* O-polysaccharide, leading to false positive test results.

In addition, the labelling of O-polysaccharide with fluorescein is poorly reproducible from the point of view of the amount of the attached fluorescein residues and their location along the polysaccharide chain. The overall disadvantages of natural O-polysaccharides led to the idea of using synthetic oligosaccharides, which mimic the M epitope of the polysaccharide chain, as diagnostic antigens. The presence of the (1→3)-bond is a unique feature of the *Brucella* O-polysaccharide, as this structural element has not been found in the O-polysaccharides of other bacteria. Thus, Bundle et al. synthesized a series of oligosaccharides representing various structural motifs within the O-polysaccharide chain of *Brucella* (Bundle and McGiven, 2017; Ganesh et al., 2014; Guiard et al., 2013). Their BSA conjugates were employed as diagnostic antigens in ELISA. Notably, short oligosaccharides demonstrated greater specificity compared to natural O-antigens (Duncombe et al., 2022). Based on this observation, the authors proposed that the epitope formed by the terminal monosaccharide unit provides higher diagnostic specificity than that formed by internal monosaccharide residues within native O-antigens.

Recently, we reported the synthesis of a series of spacered oligosaccharides featuring the presence of an α-(1→3)-glycoside bond and related to the M epitope of the *Brucella* O-polysaccharide (Tsvetkov et al., 2024; Tsvetkov and Nifantiev, 2023). Fluorescein conjugates of these compounds were used in the development of brucellosis diagnosis based on FPA (Mukhametova et al., 2024a). Fluorescein-based dyes are the most popular due to their unique spectral properties (high quantum yield of 92% and fluorescence lifetime of 4.05 ns). Fluorescein dyes are chemically stable, inexpensive and commercially available. In addition, most FP readers are equipped with filters for measuring fluorescence of fluorescein. As demonstrated, the FPA method has proven to be effective for detecting antibodies against *Brucella* in various physiological fluids – such as serum, whole blood, and milk – across different animal species and in humans (Dong et al., 2021; Ibarra et al., 2023; Olsen et al., 2025; Sotolongo-Rodríguez et al., 2022). Here we describe the synthesis of α-(1→2)-linked D-perosamine oligomers (**1a**–**4a**), corresponding to the A epitope of the *Brucella* O-polysaccharide (Figure 1), the preparation of their fluorescein-labeled conjugates (**1b**–**4b**) and the investigation of their applicability as tracers in FPA to evaluate anti-*Brucella* serum immunoglobulins and to determine the optimal tracer size for effective diagnosis of brucellosis in animals.

## Results and discussion

### Synthesis of oligosaccharides 1a-4a and fluorescein labeled conjugates 1b-4b thereof

**SCHEME 1:**
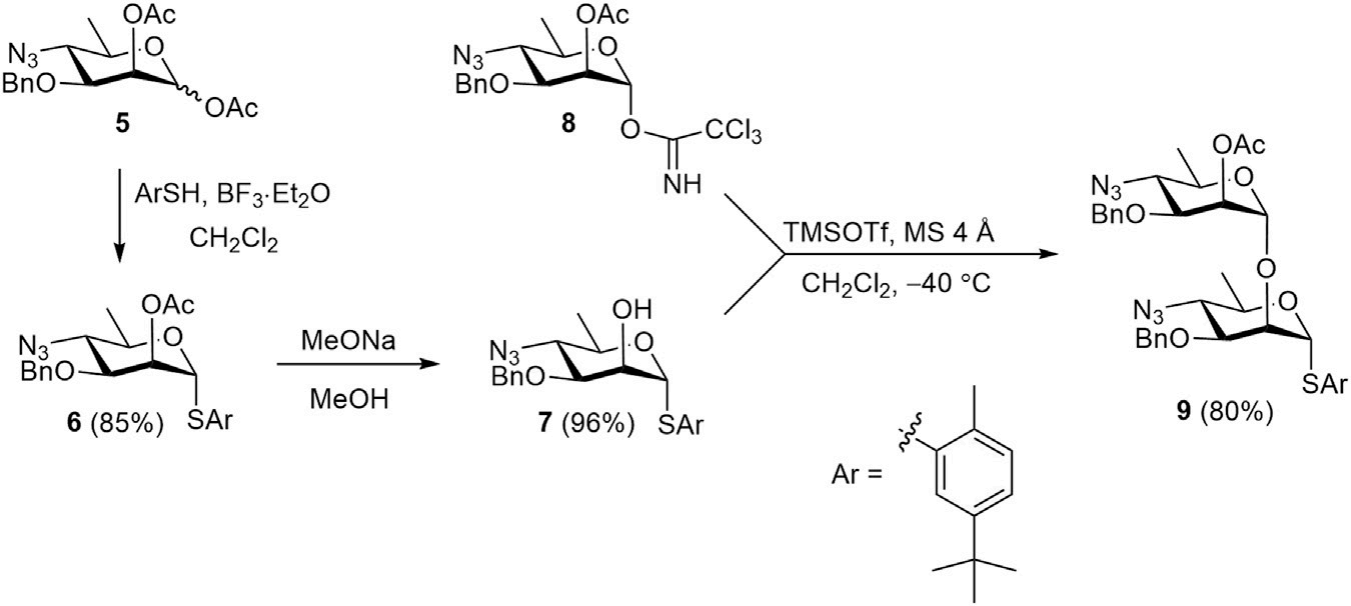
Synthesis of synthetic blocks **6**, **7**, and **9**.

The known 1,2-diacetate **5** (Bundle et al., 1988) was employed to prepare the key synthetic blocks **6**, **7**, and **9**, which were then used to assemble all protected oligosaccharides (Scheme 1). Treatment of diacetate **5** with 5-(*tert*-butyl)-2-methylthiophenol in the presence of BF_3_⋅Et_2_O smoothly produced thioglycoside **6**, which was O-deacetylated to give 2-OH derivative **7** almost quantitatively. The use of azide groups as precursors to amides is a well-established strategy in the synthesis of perosamine and structurally related anthrose oligomers (Bundle et al., 1988; Bundle and McGiven, 2017; Saksena et al., 2005; Saksena et al., 2008). The preference for azide over amide protecting groups arises from the observation that amide at the C-4 position of glycosyl donors significantly decreased the stereoselectivity of glycosylation (Adamo and Kováč, 2007). Acetyl group at C-2 atom of glycosyl donor **8** provided the required α-selectivity in glycosylation and served as a convenient temporary protecting group. Orthogonal glycosylation of acceptor **7** with imidate **8** (Ariosa-Alvarez et al., 1998) afforded disaccharide thioglycoside **9** in high yield. The latter was used for the block-wise assembly of the target (1→2)-linked oligosaccharides.

The published data evidenced (Hou and Kovác, 2010; Ogawa et al., 1996; Peters and Bundle, 1989; Saksena et al., 2005) that glycosyl donors derived from 4-azido-4,6-dideoxy-d-mannose are able to glycosylate secondary hydroxyl groups highly α-stereoselectively, even if these donors bear non-participating substituents (for example, monosaccharide residue) at O-2. At the same time, glycosylation of much more reactive primary aliphatic acceptors can lead to the formation of considerable amounts of β-anomers (Ogawa and Kovac, 1997).

**SCHEME 2:**
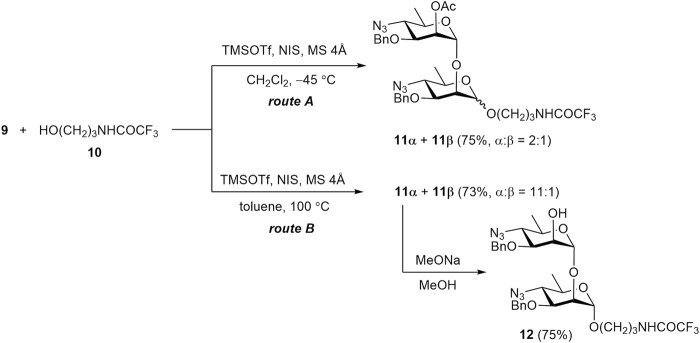
Glycosylation of 3-trifluoroacetamidopropanol **10** with donor **9** under different conditions.

Indeed, TMSOTf–NIS promoted glycosylation of acceptor **10** with donor **9** under the conditions commonly used for the thioglycoside activation (at −45 °C) resulted in a mixture of glycosides **11α** and **11β** in a ratio of 2:1 (Scheme 2, route A). To improve the selectivity of glycosylation, the reaction was carried out in toluene at elevated temperature (Scheme 2, route B). This led to a significant increase in the α-stereoselectivity of the reaction, resulting in an **11α**:**11β** ratio of 11:1. The anomeric configuration of the products was confirmed by ^1^ *J*_C1,H1_ values (170.1 Hz for **11α** and 155.7 Hz for **11β**).

The preferential formation of the thermodynamically more stable α-anomer at elevated temperature was reported by Hou and Kovác (2010) and termed “thermodynamically controlled glycosylation”. However, a detailed mechanistic explanation for this phenomenon has not been clearly addressed in the literature. The term “thermodynamic control” conventionally implies that equilibrium is established among reaction products during the course of the reaction. Therefore, we investigated the possibility of interconversion between α- and β-glycosides under the reported reaction conditions.

Although anomerization of a glycosidic bond without a leaving group is rare, several examples of such a transformation have been described in the literature (Manabe et al., 2014). In this connection, we examined whether equilibration between **11α** and **11β** really occurs under the conditions used for “thermodynamically controlled” glycosylation (*i.e.*, TMSOTf, toluene, 100 °C) (Hou and Kovác, 2010). However, when β-glycoside **11β** was thus treated with TMSOTf the expected transformation to a mixture of **11α** and **11β** was not observed. This result suggests that the concept of “thermodynamic control” may refer to the equilibrium between some intermediate products rather than the final glycosides.

Conventional deacetylation of **11α** with sodium methoxide yielded 2-OH derivative **12**. It was then used for transformation into final disaccharide **1**, and also served as a glycosyl acceptor in the synthesis of tetrasaccharide **12**.

**SCHEME 3:**
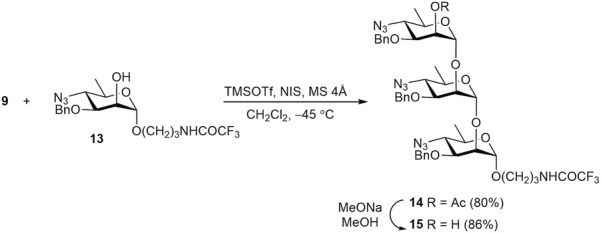
Synthesis of protected trisaccharide **14**.

NIS–TMSOTf promoted glycosylation of known acceptor **13** (Tsvetkov et al., 2024) with thioglycoside **9** smoothly gave trisaccharide **14** (Scheme 3). As expected, glycosylation of the less reactive secondary OH group in **13** exclusively produced the α-linked product **14**. Its O-deacetylation gave alcohol **15**, the precursor of target trisaccharide **2**.

**SCHEME 4:**
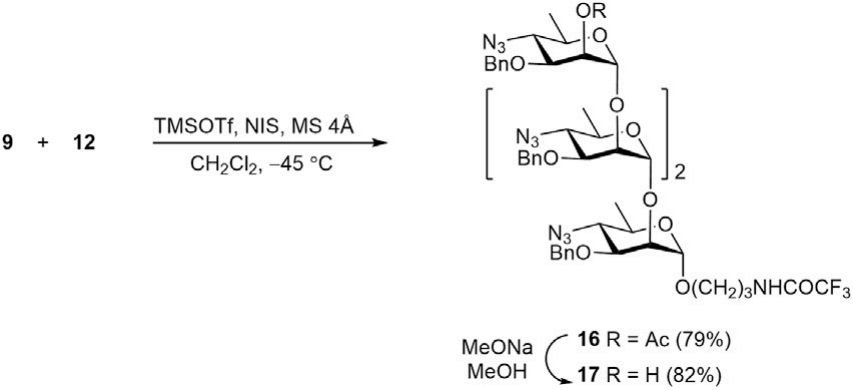
Synthesis of protected tetrasaccharide **16**.

Similarly, glycosylation of disaccharide acceptor **12** with thioglycoside **9** resulted in α-linked tetrasaccharide **16**, with no detectable formation of the corresponding β-anomer (Scheme 4). Subsequent O-deacetylation of **16** gave rise to 2-OH derivative **17**, which was then converted into tetrasaccharide **3**.

**SCHEME 5:**
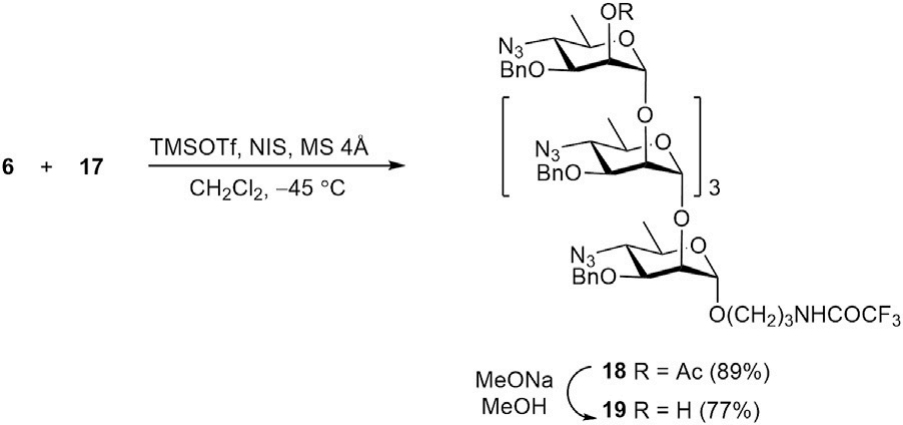
Synthesis of protected pentasaccharide **18**.

Finally, tetrasaccharide acceptor **17** was subjected to glycosylation with thioglycoside donor **6** to produce protected pentasaccharide **18** in high yield. Its O-deacetylation led to the formation of alcohol **19**, which was then transformed to free pentasaccharide **4** (Scheme 5). The structure of protected oligosaccharides **14**–**19**, particularly the configuration of all anomeric centers was confirmed by characteristic values of corresponding J_H1,H2_ constants and other data of fully assigned ^1^H and ^13^C NMR spectra (see Supplementary Material) and high-resolution mass spectra (HRMS).

**SCHEME 6:**
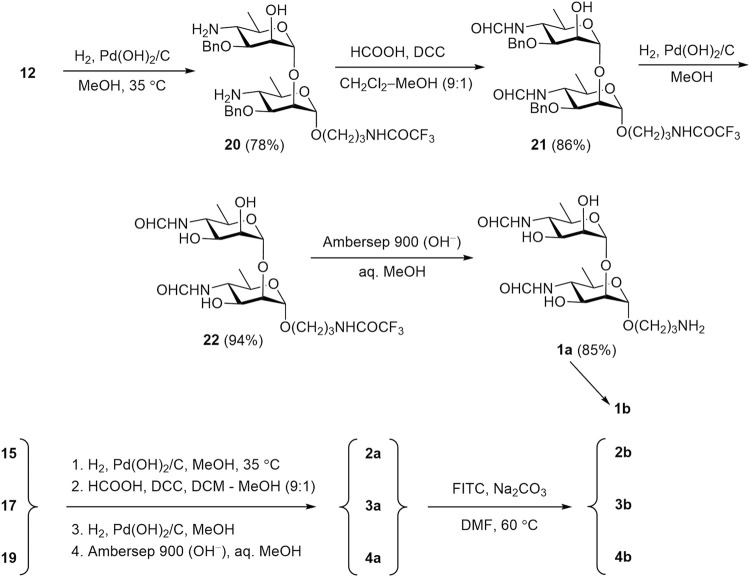
Synthesis of free oligosaccharides **1a-4a** and their conversion in fluorescein labeled conjugates **1b**-**4b**.

The transformation of protected oligomers **12**, **15**, **17**, and **19** into final products **1a**-**4a** was accomplished by using the reaction sequence described in our previous publication (Tsvetkov et al., 2024). It included reduction of azido groups by catalytic hydrogenation, N-formylation, debenzylation and removal of the N-trifluoroacetyl group. The details of this transformation are shown in Scheme 6, using disaccharide **12** as an example. Thus, catalytic reduction of diazide **12** over Pd(OH)_2_/C was accompanied with only minor affecting benzyl groups (Ariosa-Alvarez et al., 1998) and produced, after chromatographic purification, diamine **20** in 78% yield. N-Formylation of the latter with formic anhydride generated *in situ* from formic acid and DCC resulted in the formation of bis(formamide) **21** (86%). Following catalytic debenzylation of **21** afforded triol **22** (94%); final basic removal of the N-trifluoroacetyl group from **22** gave target 3-aminopropyl glycoside **1a** in 85% yield after purification by size-exclusion chromatography.

Intermediate products **20**–**22** were characterized as follows. The structure of compound **20** was confirmed by ^1^H and ^13^C NMR, and HRMS data, while the composition of N-formyl derivatives **21** and **22** was characterized by only HRMS data, since their NMR spectra were poorly informative due to the *Z*/*E*-isomerism of the N-formyl groups (Peters et al., 1990).

Protected oligomers **15**, **17**, and **19** underwent the same transformations to produce desired free 3-aminopropyl glycosides **2a**-**4a** (Scheme 6). The existence of compounds **2a**-**4a** as mixtures of 2^n^ isomers (where n is the number of the N-formyl monosaccharide units in the molecule) (Peters et al., 1990) strongly complicates NMR spectra, thus making their full assignment virtually impossible. Nevertheless, the presence of key structural features, such as N-formyl groups (both *Z*- and *E*-isomers), anomeric centers, the 3-aminopropyl spacer moiety, and some others, can be inferred from ^1^H and ^13^C NMR spectra of compounds **1a**-**4a**. Further introduction of the fluorescein tag was conducted with FITC in the presence of base (Na_2_CO_3_) in DMF. The primary amino group in the spacer reacts rapidly and regioselectively with the isothiocyanate group of FITC, leading to fluorescein-labeled tracers with good yields (80–88%).

### Assaying of serum immunoglobulins with the use of fluorescein labeled conjugates 1b-4b

*Brucella*-positive (+) (N = 19) and *Brucella*-negative (−) (N = 20) serum samples, which were validated through conventional serological methods (the Rose Bengal test (RBT), the complement fixation test (CFT) and ELISA), were used to evaluate the diagnostic performance of FPA employing the fluorescent tracers **1b**–**4b** synthesized in this work. Also, the previously reported monosaccharide tracer (compound **23**) (Mukhametova et al., 2024a) was included in this study.

**FIGURE 2:**
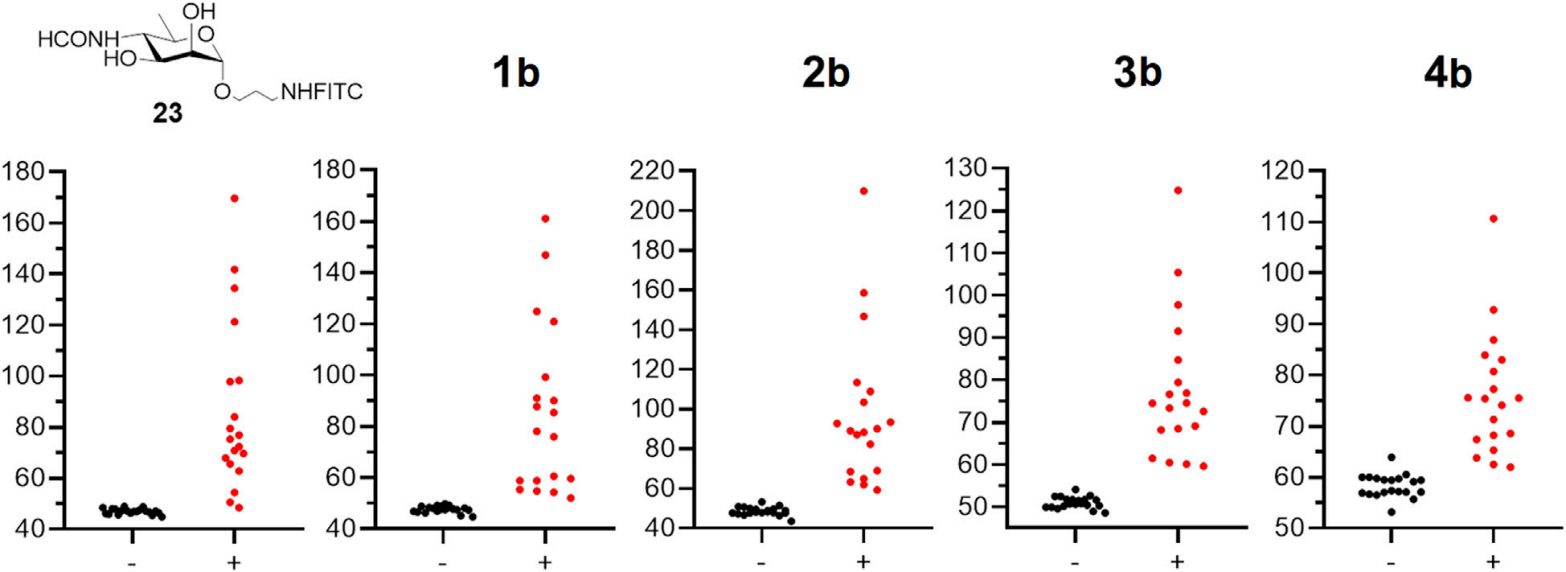
The FPA results for negative (−) (N = 20) and positive (+) (N = 19) bovine serum samples on brucellosis using tracers: **23** and **1b–4b**. All experiments were repeated three times and the mean values and standard deviations were calculated. For all samples the experimental error did not exceed 10%.

The FPA method is based on the increase in the FP signal caused by the interaction of fluorescence oligosaccharide probes with oligosaccharide-specific antibodies (Eremin et al., 2024). Accordingly, all tracers tested (**23**, **1b**–**4b**) produced a significant increase in FP signal in the *Brucella*-positive group compared to the *Brucella*-negative group (see Figure 2; Table 1). Notably, in *Brucella*-negative samples, a slight gradual increase in FP signal was observed with enlargement of oligosaccharide length–median FP values ranged from 47.0 for the monosaccharide tracer **23** to 58.3 for the pentasaccharide tracer **4b**. This trend may be attributed to nonspecific interactions between the larger oligosaccharides and various serum immunoglobulins and other proteins.

**TABLE 1:** Median FP signal values obtained by binding fluorescently labeled conjugates to negative (−) sera and *Brucella*-positive (+) bovine sera samples using tracers **23** and **1b–4b**.

Group	Median, mP				
	23	1b	2b	3b	4b
+ (N = 19)	75.3	78.1	89.1	74.5	75.4
- (N = 20)	47.0	47.7	48.3	50.8	58.3
ΔmP	28.3	30.4	40.8	23.7	17.1

**FIGURE 3:**
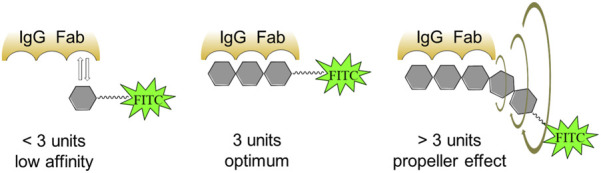
Occurrence of the propeller effect upon binding of fluorescently labeled glycoconjugates to antibodies.

To determine the specificity and sensitivity of the FPA method when using fluorescently labeled saccharides of different lengths, ROC analysis of the obtained data was performed. The results are presented in Table 2. As can be seen, the diagnostic sensitivity of the method for all synthesized tracers is comparable. However, the specificity is higher for conjugates containing from 2 to 4 perosamine units. While testing, *Brucella*-positive sera with fluorescent glycoconjugate tracers, a bell-shaped dependence was observed with a maximum increase in the FP signal in the case of trisaccharide conjugate **2b** (Table 1). It is possible that the decrease in the FP signal for **3b** and **4b** containing four and five perosamine units, respectively, can be explained by the so-called “propeller effect” (Nasir and Jolley, 2003). It is generally assumed that specific anti-*Brucella* antibodies primarily recognize epitopes located at the non-reducing end of the oligosaccharide chain. Consequently, the addition of extra sugar residues beyond the antibody binding site may increase the rotational mobility of the part bearing the fluorescent label and of the fluorescein itself (Figure 3).

**TABLE 2:** Receiver operating characteristic (ROC) analysis of sensitivity and specificity of FPA with conjugates **1b**-**4b** and **23**.

ROC values	Fluorescent glycoconjugates				
	23	1b	2b	3b	4b
ROC Curve Area	0.993	1	1	1	0.992
Cut-off, mP	48.5	50	54	56	62
sensitivity, %	100	100	100	100	100
specificity, %	85	100	100	100	95

To interpret this phenomenon, a “wobbling-in-a-cone” model has been proposed (Kinosita et al., 1982; Palchowdhury et al., 2024), which suggests that the fluorophore undergoes restricted rotational motion within an imaginary cone. It is known that FP measures the alignment of emitted light relative to polarized excitation, which depends on the tracer’s rotation during its fluorescence lifetime (*τ*). Rotation angle (θ) is the angular displacement of the tracer during *τ*. Increase in θ leads to a faster rotation and lower polarization (*P*). In FP, the rotation angle of the tracer depends on the rotational diffusion, flexibility of the tracer and the hydrodynamic radius of the complex (tracer-antibody). When the tracer binds to the antibody, the FP signal increases. However, the signal increase is also affected by the size of the tracer itself. As can be seen, binding of a short tracer (2–3 saccharides) to the antibody leads to a sharp slowdown in its rotation and an increase in the FP signal is observed. Growth in numbers of monosaccharide units in the tracer glycoconjugates increases θ, since the distance between the fluorophore and the antibody-antigen complex expands, thereby multiplying the freedom of movement of the fluorophore and leading to a corresponding decrease in the FP signal. A similar effect was described in works on the design of tracers for the detection of low-molecular toxicants by FPIA (Dong et al., 2019).

Thus, as shown in Table 1, the maximum difference in median FP signals between *Brucella*-positive and *Brucella*-negative sera was observed for conjugate **2b**, which contains three monosaccharide units. Varying of the oligosaccharide length influenced the FP signal difference. Therefore, the trisaccharide represents the most efficient size for use as a fluorescent tracer in FPA for the detection of anti-*Brucella* antibodies. It should be mentioned that the advantage of synthetic oligosaccharide antigens, which permits better distinguishing of anti-*Brucella* and anti-*Y. enterocolitica* O:9, was reported (Duncombe et al., 2022). Overall the obtained results can form the basis for the elaboration of advantageous serological tests for brucellosis.

## Conclusion

In conclusion, 3-aminopropyl glycosides of α-(1→2)-linked oligomers of 4,6-dideoxy-4-formamido-d-mannopyranose up to the pentasaccharide, which represent the A epitope of the *Brucella* O-polysaccharide, have been successfully synthesized using a block-wise approach. The α-(1→2)-linked disaccharide thioglycoside **9** was employed as the key donor block for the assembly of the required oligomers. Despite the presence of a non-participating glycosyl substituent at O-2 of the donor molecule, glycosylation of the 2-OH groups in mono- and oligosaccharide acceptors demonstrated high α-stereoselectivity. On the contrary, glycosylation of the more reactive primary aliphatic alcohol, 3-trifluroacetamidopropanol, with the same donor resulted in a significant decrease in α-stereoselectivity. It was considerably improved by performing the reaction under the conditions of “thermodynamic control”, *i.e.*, at elevated (100 °C) temperature. However, a control experiment showed that interconversion of the anomeric glycosides did not occur under these conditions, and thus the improvement in α-stereoselectivity is a more complex process than simply achieving an equilibrium state.

The synthesized α-(1→2)-linked oligomers were transformed into corresponding fluorescein labelled conjugates, which were applied as tracers for assaying anti-*Brucella* immunoglobulins by fluorescence polarization. This study demonstrated that the trisaccharide derivative is the most efficient for detecting antibodies against *Brucella* by the FPA. It is important to point out that the use of longer oligosaccharides gave a smaller fluorescence polarization signal that is explained by us by the existence of known “propeller-effect.” This finding shows again the advantage of synthetic fluorescein-labelled oligosaccharide tracers of distinct structure when compared with fluorescein conjugates of *Brucella* O-polysaccharide, which are used often today as tracers for FPA but contain poorly characterized amounts of label attached along the polysaccharide chains. Thus, fluorescently labeled α-(1→2)-linked trisaccharide exhibits the best properties among the tested compounds and permits the most efficient detecting of antibodies to *Brucella.* Thus, this tracer can be recommended for the development of diagnostic tools to identify brucellosis in animals and humans.

## Experimental section

### General methods

NMR spectra were recorded on a Bruker Fourier 300 HD and Bruker Avance 600 NMR spectrometers. Protected oligosaccharides were measured in CDCl_3_, and ^1^H NMR chemical shifts were referenced to the solvent residual signal (δ_H_ 7.27). ^13^C chemical shifts were referenced to the central resonance of CDCl_3_ (δC 77.0). Free oligosaccharides were measured in deuterium oxide (D_2_O) with suppression of the HOD signal. Acetone (δ_H_ 2.225, δ_C_ 31.45) was used as an internal standard. Signal assignment was made using COSY, TOCSY, HSQC, and ROESY experiments. In the presentation of NMR data, monosaccharide residues in oligosaccharides are denoted by the capital letters (A, B, C, *etc.*) starting from the reducing end. NMR spectra of synthesized compounds are presented in the Supplementary Material. High resolution mass spectrometry (HRMS) with electrospray ionization (ESI) was performed on a MicrOTOF II (Bruker Daltonics) instrument. Optical rotations were measured using a JASCO P-2000 polarimeter at ambient temperature (20 °C–25 °C) in chloroform (protected oligosaccharides) or water (free oligosaccharides). TLC was performed on Silica Gel 60 F254 plates (Merck) and visualization was accomplished using UV light or by charring at ∼150 °C with orcinol–phosphoric acid [180 mg of orcinol in a mixture of 85% H_3_PO_4_ (10 mL), ethanol (5 mL), and water (85 mL)]. Column chromatography was carried out using Silica Gel 60 (40–63 μm; Merck Millipore). Gel-permeation chromatography of free oligosaccharides was performed on a Toyopearl TSK HW-40(S) column (2.8 × 80 cm) in 0.1 M acetic acid. A K-2401 refractive index detector (Knauer) was used to monitor gel-permeation chromatography. All moisture-sensitive reactions were carried out using dry solvents under dry argon. Powdered molecular sieve 4 Å was activated at 300 °C under vacuum (∼1 mbar) for 30 min directly prior to the reaction.

**5-*tert*-Butyl-2-methylphenyl 2-*O*-acetyl-4-azido-3-*O*-benzyl-4,6-dideoxy-1-thio-α-d-mannopyranoside (6)**. 5-*tert*-Butyl-2-methylthiophenol (0.89 g, 4.92 mmol) and BF_3_⋅Et_2_O (0.49 mL, 3.93 mmol) were added to a chilled (ice bath) solution of 1,2-diacetate **5** (1.22 g, 3.28 mmol) in CH_2_Cl_2_ (30 mL), and the mixture was stirred for 10 min with chilling and then for 2.5 h at room temperature. The mixture was diluted with CHCl_3_ (70 mL), washed with water (80 mL) and aqueous saturated NaHCO_3_. The organic solution was concentrated, and residue was purified by column chromatography (petroleum ether–EtOAc, 0→5%) to yield thioglycoside **6** (1.38 g, 85%) as a colorless syrup, [α]_D_ +116.2 (c 1.0, CHCl_3_); the product contained 8%–9% of the β-anomer. ^1^H NMR (CDCl_3_, 300 MHz): δ 7.56–7.12 (m, 8 H, Ar), 5.65 (dd, 1 H, *J*_2,1_ = 1.6 Hz, *J*_2.3_ = 3.1 Hz, H-2), 5.37 (d, 1 H, *J*_1,2_ = 1.6 Hz, H-1), 4.76 (d, 1 H, *J* = 11.0 Hz, PhC*Ha*Hb), 4.61 (d, 1 H, *J* = 11.0 Hz, PhCHa*Hb*), 4.11 (dq, 1 H, *J*_5,6_ = 6.2 Hz, *J*_5,4_ = 10.0 Hz, H-5), 3.87 (dd, 1 H, *J*_3,2_ = 3.1 Hz, *J*_3,4_ = 10.1 Hz, H-3), 3.54 (t, 1 H, *J* = 10.0 Hz, H-4), 2.42 (s, 3 H, Ar-CH_3_), 2.16 (s, 3H, CH_3_CO), 1.38 (d, 3 H, *J*_6,5_ = 6.2 Hz, H-6), 1.33 (s, 9 H, C(CH_3_)_3_). ^13^C NMR (76 MHz, CDCl_3_): δ 170.2 (CH_3_ *C*O), 149.9, 137.0, 136.7, 132.2, 130.1, 129.8, 128.5, 128.4, 128.1, 125.2 (Ar), 85.7 (C-1), 71.8 (Ph*C*H_2_), 69.4 (C-2), 68.3 (C-5), 64.2 (C-4), 34.5 (*C*(CH_3_)_3_), 31.3 (C(*C*H_3_)_3_), 21.0 (*C*H_3_CO), 20.3 (Ar-*C*H_3_), 18.5 (C-6). HRMS (ESI): calcd. for C_26_H_33_N_3_O_4_S [M + K]^+^ *m/z* 522.1819; found *m/z* 522.1823.

**5-*tert*-Butyl-2-methylphenyl 4-azido-3-*O*-benzyl-4,6-dideoxy-1-thio-α-d-mannopyranoside (7)**. 1 M Sodium methoxide in MeOH (0.47 mL) was added to a solution of 2-acetate **6** (229 mg, 0.47 mmol) in MeOH (5 mL), and the mixture was stirred for 16 h at room temperature. The mixture was neutralized with AcOH, and the solvent was evaporated. The residue was dissolved in CHCl_3_ (25 mL), and the solution was washed with water (20 mL). The aqueous phase was extracted with CHCl_3_ (25 mL), and the combined organic solutions were concentrated. Column chromatography (petroleum ether–EtOAc, 5→10%) produced title product **7** (201 mg, 96%) as a colorless syrup, [α]_D_ +201.6 (с 1, CHCl_3_). ^1^H NMR (CDCl_3_, 300 MHz): δ 7.61–7.13 (m, 8 H, Ar), 5.53 (br. s, 1H, H-1), 4.83–4.72 (m, 2 H, PhC*H*_*2*_), 4.30 (br. s, 1 H, H-2), 4.09 (dq, 1 H, *J*_5,6_ = 6.2 Hz, *J*_5,4_ = 10.1 Hz, H-5), 3.73 (dd, 1H, *J*_3,4_ = 9.7 Hz, *J*_3,2_ = 3.1 Hz, H-3), 3.54 (t, 1 H, *J* = 9.9 Hz, H-4), 2.67 (br. s, 1 H, 2-OH), 2.39 (s, 1 H, Ar-CH_3_), 1.36 (d, 3 H *J*_6,5_ = 6.2 Hz, H-6), 1.32 (s, 9 H, C(CH_3_)_3_). ^13^C NMR (76 MHz, CDCl_3_): δ 149.9, 137.0, 136.2, 132.5, 130.0, 129.1, 128.7, 128.4, 128.2, 124.8 (Ar) 86.4 (C-1), 78.7 (C-3), 72.3 (Ph*C*H_2_), 69.1 (C-2), 68.0 (C-5), 64.3 (C-4), 34.5 (*C*(CH_3_)_3_), 31.3 (C(*C*H_3_)_3_), 20.2 (Ar-*C*H_3_), 18.4 (C-6). HRMS (ESI): calcd. for C_24_H_31_N_3_O_3_S [M + Na]^+^ *m/z* 464.1966; found *m/z* 464.1978.

**5-*tert*-Butyl-2-methylphenyl 2-*O*-acetyl-4-azido-3-*O*-benzyl-4,6-dideoxy-α-d-mannopyranosyl-(1→2)-4-azido-3-*O*-benzyl-4,6-dideoxy-1-thio-α-d-mannopyranoside (9)**. A mixture of imidate **8** (181 mg, 0.389 mmol) and thioglycoside acceptor **7** (159 mg, 0.353 mmol) was dried by co-evaporation with toluene, dried under vacuum for 1 h, and dissolved in CH_2_Cl_2_ (5 mL). Powdered molecular sieve 4 Å (350 mg) was added to the solution, and the mixture was stirred for 30 min at room temperature and cooled to −40 °C. TMSOTf (14 μL, 0.078 mmol) was added and the mixture was stirred for 30 min, while the temperature was gradually increased to −10 °C. Stirring was continued at this temperature for another 1 h and then the reaction was quenched by adding Et_3_N (20 μL). The solids were filtered off through a Celite layer, washed with CHCl_3_ (4 × 5 mL), and the filtrate was washed with water (20 mL). The solvent was evaporated and the residue was purified by column chromatography (petroleum ether–EtOAc, 0→7%) to afford disaccharide **9** (212 mg, 80%) as a colorless syrup, [α]_D_ +130.8 (с 1, CHCl_3_). ^1^H NMR (CDCl_3_, 300 MHz): δ 7.53–7.13 (m, 13 H, Ar), 5.41 (dd, 1 H, *J*_2,1_ = 1.8 Hz, *J*_2,3_ = 3.2 Hz, H-2^B^), 5.31 (d, 1 H, *J*_1,2_ = 1.6 Hz, H-1^A^), 4.84 (d, 1 H, *J*_1,2_ = 1.6 Hz, H-1^B^), 4.85–4.53 (m, 4 H, 2 PhC*H*_*2*_), 4.12 (poorly resolved t, 1 H, H-2^A^), 4.02 (dq, 1 H, *J*_5,4_ = 10.1 Hz, *J*_5,6_ = 6.2 Hz, H-5^B^), 3.83–3.76 (m, 2 H, H-3^A^, H-3^B^), 3.61 (dq, 1 H, *J*_5,4_ = 10.1 Hz, *J*_5,6_ = 6.2 Hz, H-5^A^), 3.48–3.36 (m, 2 H, H-4^A^, H-4^B^), 2.38 (s, 3 H, Ar-CH_3_), 2.13 (s, 3 H, CH_3_CO), 1.35–1.30 (m, 12 H, H-6^B^, C(CH_3_)_3_), 1.23 (d, 3 H, *J*_6,5_ = 6.2 Hz, H-6^A^). ^13^C NMR (76 MHz, CDCl_3_): δ 169.9 (CH_3_ *C*O), 149.9, 137.4, 137.1, 136.5, 132.6, 130.1, 129.7, 128.6, 128.5, 128.1, 125.2 (Ar), 99.7 (C-1^A^), 86.9 (C-1^B^), 78.2 (C-3^B^), 76.4 (C-2^A^), 75.3 (C-3^A^), 72.2, 71.6 (2 Ph*C*H_2_), 68.4 (C-5^B^), 67.7 (C-5^A^), 67.2 (C-2^B^), 64.3 (C-4^B^), 63.8 (C-4^A^), 31.3 (C(*C*H_3_)_3_), 21.0 (*C*H_3_CO), 20.2 (Ar-*C*H_3_), 18.5 (C-6^B^), 18.4 (C-6^A^). HRMS (ESI): calcd. for C_39_H_48_N_6_O_7_S [M + Na]^+^ *m/z* 767.3197; found *m/z* 767.3200.

**3-Trifluoroacetamidopropyl 2-*O*-acetyl-4-azido-3-*O*-benzyl-4,6-dideoxy-α-d-mannopyranosyl-(1→2)-4-azido-3-*O*-benzyl-4,6-dideoxy-α- and β-d-mannopyranosides (11α, 11β)**.

1. A mixture of donor **9** (100 mg, 0.134 mmol), alcohol **10** (34.5 mg, 0.202 mmol) and mol. sieve 4 Å (150 mg) in CH_2_Cl_2_ (3 mL) was stirred for 30 min at room temperature, and then was cooled to −45 °C. NIS (60.3 mg, 0.268 mmol) was added, the mixture was stirred for 30 min, and then TMSOTf (5 μL, 0.027 mmol) was added. The mixture was stirred for 40 min, after which more TMSOTf (7 μL, 0.038 mmol) was added. The resulting mixture was stirred for 4 h, gradually increasing the temperature to −10 °C. The reaction was quenched by adding pyridine (30 μL), the mixture was diluted with CHCl_3_ (10 mL), the mol. sieve was filtered off through a Celite layer, and washed with CHCl_3_ (3 × 5 mL). The filtrate was washed with 0.5 M Na_2_S_2_O_3_ solution (20 mL) and water (30 mL), and concentrated. The solvent was evaporated and the residue was subjected to column chromatography (toluene – EtOAc, 0→15%) to provide a mixture of glycosides **11α** and **11β** (70 mg, 75%) in a ratio ∼2:1 (NMR data).
2. A solution of donor **9** (996 mg, 1.34 mmol) in toluene (45 mL) was added to alcohol **10** (404 mg, 2.36 mmol) and the mixture was heated to 100 °C. Mol. sieve 4 Å (1.5 g), NIS (608 mg, 2.70 mmol), and a solution of TMSOTf (49 μL) in toluene (5 mL) were added and the mixture was stirred at 100 °C. Additional portions of the promoting reagents were added after 2 h (304 mg (1.35 mmol) of NIS; 25 μL (0.14 mmol) of TMSOTf) and 4 h (152 mg of NIS; 12 μL of TMSOTf). Stirring was continued at 100 °C for another 1 h and then the mixture was cooled to room temperature, and quenched by adding pyridine (200 μL). The mol. sieve was filtered off through a Celite layer and washed with toluene (3 × 10 mL). The filtrate was washed with 0.5 M Na_2_S_2_O_3_ solution (50 mL) and water (50 mL), and concentrated. Column chromatography (petroleum ether–EtOAc, 10→25%) of the residue gave α-glycoside **11α** (472 mg), β-glycoside **11β** (24 mg), and 310 mg of their mixture. The latter mixture was rechromatographed to produce more **11α** (186 mg) and **11β** (47 mg). Total yields of **11α** and **11β** were 658 mg (67%) and 71 mg (6%), respectively.

α-Anomer **11α**, white crystals, m. p. 111°C–113°C, [α]_D_ +78.3 (с 1, CHCl_3_). ^1^H NMR (CDCl_3_, 300 MHz): δ 7.44–7.26 (m, 10 H, Ar), 6.68 (br. s, 1 H, NH), 5.42 (dd, 1 H, *J*_2,1_ = 1. 9 Hz, *J*_2,3_ = 3.3 Hz, H-2^B^), 4.89 (d, 1 H, *J*_1,2_ = 1.9 Hz, H-1^B^), 4.78–4.68 (m, 3 H, H-1^A^, 2 PhC*Ha*Hb), 4.63 (d, 1 H, *J* = 11.5 Hz, PhCHa*Hb*), 4.55 (d, 1 H, *J* = 11.1 Hz, PhCHa*Hb*), 3.88 (poorly resolved dd, 1 H, H-2^A^), 3.83–3.73 (m, 2 H, H-3^B^, OC*Ha*HbCH_2_CH_2_N), 3.70 (dd, 1 H, *J*_3,2_ = 2.9 Hz, *J*_3,4_ = 9.3 Hz, H-3^A^), 3.65–3.33 (m, 7 H, H-4^A^, H-4^B^, H-5^A^, H-5^B^, OCHa*Hb*CH_2_CH_2_N, OCH_2_CH_2_C*H*_*2*_N), 2.10 (s, 3 H, CH_3_CO), 1.94–1.83 (m, 2 H, OCH_2_C*H*_*2*_CH_2_N), 1.36–1.29 (m, 6 H, H-6^A^, H-6^B^). ^13^C NMR (76 MHz, CDCl_3_): δ 169.8 (CH_3_ *C*O), 137.4, 137.1, 128.5, 128.5, 128.1, 128.0, 127.8 (Ar), 99.4 (C-1^B^), 98.9 (C-1^A^), 77.8 (C-3^A^), 75.3 (C-3^B^), 73.6 (C-2^A^), 72.1, 71.6 (2 Ph*C*H_2_), 67.7 (C-5^B^), 67.4 (C-5^A^), 67.1 (C-2^B^), 65.8 (O*C*H_2_CH_2_CH_2_N), 64.0 (C-4^B^), 63.8 (C-4^A^), 38.1 (OCH_2_CH_2_ *C*H_2_N), 28.4 (OCH_2_ *C*H_2_CH_2_N), 21.0 (*C*H_3_CO) 18.6 (C-6^A^), 18.5 (C-6^B^). HRMS (ESI): calcd. for C_33_H_40_F_3_N_7_O_9_ [M + K]^+^ *m/z* 774.2471; found *m/z* 774.2472.

β-Anomer **11β**, colorless syrup, [α]_D_ +9.9 (с 1, CHCl_3_). ^1^H NMR (CDCl_3_, 300 MHz): δ 7.42–7.27 (m, 10 H, Ar), 7.13 (br. s, 1 H, NH), 5.49 (dd, 1 H, *J*_2,1_ = 1.9 Hz, *J*_2,3_ = 3.3 Hz, H-2^B^), 5.09 (d, 1 H, *J*_1,2_ = 1.9 Hz, H-1^B^), 4.77–4.65 (m, 3 H, 2 PhC*Ha*Hb, PhCHa*Hb*), 4.51 (d, 1 H, *J* = 11.1 Hz, PhCHa*Hb*), 4.33 (br. s, 1 H, H-1^A^), 4.13 (poorly resolved d, 1 H, H-2^A^), 4.00 (dq, 1 H, *J*_5,4_ = 10.3 Hz, *J*_5,6_ = 6.2 Hz, H-5^B^), 3.87–3.78 (m, 2 H, H-3^B^, OC*Ha*HbCH_2_CH_2_N), 3.66–3.58 (m, 1 H, OCHa*Hb*CH_2_CH_2_N), 3.52–3.26 (m, 5 H, H-3^A^, H-4^A^, H-4^B^, OCH_2_CH_2_C*H*_*2*_N), 3.21–3.11 (m, 1 H, H-5^A^), 2.10 (s, 3 H, CH_3_CO), 1.96–1.71 (m, 2 H, OCH_2_C*H*_*2*_CH_2_N), 1.39 (d, 3H, *J*_6,5_ = 6.1 Hz, H-6^A^), 1.30 (d, 3 H, *J*_6,5_ = 6.2 Hz, H-6^B^). ^13^C NMR (76 MHz, CDCl_3_): δ 169.9 (CH_3_ *C*O), 137.2, 136.9, 128.6, 128.4, 128.1, 128.0, 127.8 (Ar) 99.5 (C-1^A^), 98.1 (C-1^B^), 80.5 (C-3^A^), 75.4 C-3^B^), 71.9 (Ph*C*H_2_), 71.4 (×2) (C-5^A^, Ph*C*H_2_), 70.7 (C-2^A^), 67.0 (C-2^B^), 66.9 (C-5^B^), 66.6 (O*C*H_2_CH_2_CH_2_N), 64.0 (C-4^B^), 63.9 (C-4^A^), 37.0 (OCH_2_CH_2_ *C*H_2_N), 28.6 (OCH_2_ *C*H_2_CH_2_N), 21.0 (*C*H_3_CO), 18.4 (×2) (C-6^A^, C-6^B^).). HRMS (ESI): calcd. for C_33_H_40_F_3_N_7_O_9_ [M + Na]^+^ *m/z* 758.2753; found *m/z* 758.2732.

**An attempt to transform 11β into an equilibrium mixture of 11β and 11α**. A solution of TMSOTf in toluene (0.03 M, 43 μL) was added at 100 °C to a stirred mixture of glycoside **11β** (19 mg, 0.026 mmol) in toluene (1 mL) and mol. sieve 4 Å (43 mg). Stirring was continued at 100 °C, and the reaction was monitored by TLC in petroleum ether–EtOAc (7:3). Starting glycoside **11β** had R_*f*_ = 0.29 in the specified solvent system. Comparison with an authentic sample of **11α** (R_*f*_ = 0.36) showed that the reaction mixture contained only **11β** after 5.5 h of heating, with no formation of **11α**.

**3-Trifluoroacetamidopropyl 4-azido-3-*O*-benzyl-4,6-dideoxy-α-d-mannopyranosyl-(1→2)-4-azido-3-*O*-benzyl-4,6-dideoxy-α-d-mannopyranoside (12)**. 1 M Sodium methoxide in MeOH (157 μL) was added to a solution of acetylated disaccharide **11α** (576 mg, 0.84 mmol) in MeOH (16 mL), the mixture was stirred for 4 h at room temperature, and then made neutral by adding Amberlite IR-120 (H+). The resin was filtered off, washed with MeOH (3 × 5 mL), and the combined filtrate and washings were concentrated. Column chromatography of the residue (toluene – EtOAc, 0→20%) yielded deacetylated product **12** (438 mg, 75%) as a colorless syrup, [α]_D_ +84.1 (с 1, CHCl_3_). ^1^H NMR (CDCl_3_, 300 MHz): δ 7.46–7.16 (m, 10 H, Ar), 6.66 (br. s, 1 H, NH), 4.96 (d, 1 H, *J*_1,2_ = 1.3 Hz, H-1^B^), 4.78–4.58 (m, 5 H, H-1^A^, 2 PhC*H*_*2*_), 4.00 (br. s, 1 H, H-2^B^), 3.91 (poorly resolved t, 1 H, H-2^A^), 3.82–3.29 (m, 10 H, H-3^A^, H-3^B^, H-4^A^, H-4^B^, H-5^A^, H-5^B^, OC*H*_*2*_CH_2_CH_2_N, OCH_2_CH_2_C*H*_*2*_N), 2.35 (br. s, 1 H, OH), 1.95–1.82 (m, 2 H, OCH_2_C*H*_*2*_CH_2_N), 1.38–1.25 (m, 6 H, H-6^B^, H-6^A^). ^13^C NMR (76 MHz, CDCl_3_): δ 137.1, 128.7, 128.6, 128.4, 128.3, 128.1, 128.0 (Ar) 101.0 (C-1^B^), 99.1 (C-1^A^), 77.9 (C-3^A^), 77.5 (C-3^B^), 73.7 (C-2^A^), 72.2, 72.1 (2 Ph*C*H_2_), 67.4 (C-5^A^), 67.4 (C-5^B^), 67.1 (C-2^B^), 65.9 (O*C*H_2_CH_2_CH_2_N), 64.1 (C-4^A^), 63.8 (C-4^B^), 38.1 (OCH_2_CH_2_ *C*H_2_N), 28.4 (OCH_2_ *C*H_2_CH_2_N), 18.6 (C-6^A^), 18.4 (C-6^B^). HRMS (ESI): calcd. for C_31_H_38_F_3_N_7_O_8_ [M + K]^+^ *m/z* 732.2366; found *m/z* 732.2364.

**3-Trifluoroacetamidopropyl 2-*O*-acetyl-4-azido-3-*O*-benzyl-4,6-dideoxy-α-d-mannopyranosyl-(1→2)-4-azido-3-*O*-benzyl-4,6-dideoxy-α-d-mannopyranosyl-(1→2)-4-azido-3-*O*-benzyl-4,6-dideoxy-α-d-mannopyranoside (14)**. A mixture of donor **9** (76 mg, 0.102 mmol), acceptor **13** (40 mg, 0.093 mmol), and mol. sieve 4 Å (140 mg) in CH_2_Cl_2_ (4 mL) was stirred for 30 min. at room temperature, and then cooled to −35 °C. NIS (42 mg, 0.186 mmol) and a solution of TMSOTf (3.4 μL, 0.019 mmol) in CH_2_Cl_2_ (0.1 mL) were added and the mixture was stirred for 40 min, while the temperature was gradually increased to −10 °C. The reaction was quenched with pyridine (20 μL), the mixture was diluted with CHCl_3_ (20 mL), the solids were filtered off through a Celite layer and washed with CHCl_3_ (3 × 5 mL). The filtrate was washed with 0.5 M Na_2_S_2_O_3_ solution (20 mL) and water (20 mL), and concentrated. Column chromatography of the residue (petroleum ether–EtOAc, 5→25%) produced trisaccharide **14** (74 mg, 80%) as a colorless syrup, [α]_D_ +85.1 (с 1, CHCl_3_). ^1^H NMR (600 MHz, CDCl_3_): δ 7.41–7.34 (m, 15 H, Ar), 6.63 (br. s, 1 H, NH), 5.41 (dd, 1 H, *J*_2,1_ = 1.7 Hz, *J*_2,3_ = 3.1 Hz, H-2^C^), 4.97 (d, 1 H, *J*_1,2_ = 1.8 Hz, H-1^B^), 4.82 (d, 1 H, *J*_1,2_ = 1.7 Hz, H-1^C^), 4.74–4.53 (m, 7 H, 3 PhC*H*_*2*_, H-1^A^), 3.87 (poorly resolved t, 1 H, H-2^B^), 3.84 (poorly resolved t, 1 H, H-2^A^), 3.79–3.71 (m, 3 H, H-3^C^, OC*Ha*HbCH_2_CH_2_N, H-3^B^), 3.66 (dd, 1 H, *J*_3,4_ = 9.9 Hz, *J*_3,2_ = 2.9 Hz H-3^A^), 3.56–3.35 (m, 8 H, H-4^B^, H-4^C^, H-5^A^, H-5^B^, H-5^C^, OCHa*Hb*CH_2_CH_2_N, OCH_2_CH_2_C*H*_*2*_N), 3.26 (t, 1 H, *J* = 10.0 Hz, H-4^A^), 2.13 (s, 3 H, CH_3_CO), 1.90–1.84 (m, 2 H, OCH_2_C*H*_*2*_CH_2_N)), 1.33–1.26 (m, 6 H, H-6^A^, H-6^B^), 1.20 (d, 3 H, *J*_6,5_ = 6.2 Hz, H-6^C^). ^13^C NMR (151 MHz, CDCl_3_): δ 169.8 (CH_3_ *C*O), 137.4, 137.2, 137.1, 128.6, 128.5, 128.5, 128.2, 128.1, 128.0 (Ar), 100.4 (C-1^B^), 99.2 (C-1^C^), 99.0 (C-1^A^), 77.6 (C-3^A^), 76.7 (C-3^B^), 75.4 (C-3^C^), 73.8 (C-2^B^, C-2^A^), 72.2, 72.0, 71.6 (3 Ph*C*H_2_), 67.9 (C-5^B^), 67.7 (C-5^C^), 67.5 (C-5^A^), 67.2 (C-2^C^), 65.8 (O*C*H_2_CH_2_CH_2_N), 64.2 (C-4^A^), 64.0 (C-4^B^), 63.8 (C-4^C^), 38.2 (OCH_2_CH_2_ *C*H_2_N), 28.4 (OCH_2_ *C*H_2_CH_2_N), 21.0 (*C*H_3_CO), 18.6 (C-6^A^), 18.6 (C-6^B^), 18.4 (C-6^C^). HRMS (ESI): calcd. for C_46_H_55_F_3_N_10_O_12_ [M + Na]^+^ *m/z* 1019.3845; found *m/z* 1019.3839.

**3-Trifluoroacetamidopropyl 4-azido-3-*O*-benzyl-4,6-dideoxy-α-d-mannopyranosyl-(1→2)-4-azido-3-*O*-benzyl-4,6-dideoxy-α-d-mannopyranosyl-(1→2)-4-azido-3-*O*-benzyl-4,6-dideoxy-α-d-mannopyranoside (15)**. 1 M Sodium methoxide in MeOH (48 μL) was added to a solution of 2-acetate **14** (239 mg, 0.24 mmol) in MeOH (4.8 mL), the mixture was stirred at room temperature for 5 h, and then made neutral by adding Amberlite IR-120 (H^+^). The resin was filtered off, washed with MeOH (3 × 5 mL), and the filtrate was concentrated. Column chromatography of the residue (toluene–EtOAc, 0→15%) gave deacetylated trisaccharide **15** (196 mg, 86%) as a colorless foam, [α]_D_ +85.4 (с 1, CHCl_3_). ^1^H NMR (400 MHz, CDCl_3_): δ 7.42–7.26 (m, 15 H, Ar), 6.55 (br. s, 1 H, NH), 4.96 (d, 1 H, *J*_1,2_ = 1.7 Hz, H-1^B^), 4.92 (d, 1 H, *J*_1,2_ = 1.5 Hz, H-1^C^), 4.74–4.58 (m, 7 H, 3 PhC*H*_*2*_, H-1^A^), 3.97 (poorly resolved t, 1 H, H-2^C^), 3.90 (poorly resolved t, 1 H, H-2^B^), 3.81 (poorly resolved t, 1 H, H-2^A^), 3.77–3.62 (m, 4 H, OC*Ha*HbCH_2_CH_2_N, H-3^B^, H-3^C^, H-3^A^), 3.55–3.34 (m, 7 H, H-4^C^, H-5^A^, H-5^B^, H-5^C^, OCHa*Hb*CH_2_CH_2_N, OCH_2_CH_2_C*H*_*2*_N), 3.31 (t, 1 H, *J* = 10.0 Hz, H-4^B^), 3.24 (t, 1 H, *J* = 9.8 Hz, H-4^A^), 2.28 (br. s, 1 H, OH) 1.88–1.80 (m, 2 H, OCH_2_C*H*_*2*_CH_2_N), 1.31–1.25 (m, 6 H, H-6^A^, H-6^B^), 1.18 (d, 3 H, *J*_6,5_ = 6.1 Hz, H-6^C^). ^13^C NMR (101 MHz, CDCl_3_): δ 137.3, 137.2, 129.0, 128.6, 128.3, 128.2, 128.1, 128.1 (Ar) 100.6 (×2) (C-1^B^, C-1^C^), 99.0 (C-1^A^), 77.6 (C-3^A^), 77.5 (C-3^C^), 76.8 (C-3^B^), 73.7 (C-2^A^), 73.4 (C-2^B^), 72.2, 72.14, 72.07 (3 Ph*C*H_2_), 67.9 (C-5^B^), 67.5 (C-5^A^), 67.4 (C-5^C^), 67.2 (C-2^C^), 65.8 (O*C*H_2_CH_2_CH_2_N), 64.4 (C-4^A^), 64.2 (C-4^B^), 63.8 (C-4^C^), 38.0 (OCH_2_CH_2_ *C*H_2_N), 28.4 (OCH_2_ *C*H_2_CH_2_N), 18.6 (C-6^A^), 18.5 (C-6^C^), 18.3 (C-6^B^). HRMS (ESI): calcd. for C_44_H_53_F_3_N_10_O_11_ [M + K]^+^ *m/z* 993.3479; found *m/z* 993.3475.

**3-Trifluoroacetamidopropyl 2-*O*-acetyl-4-azido-3-*O*-benzyl-4,6-dideoxy-α-d-mannopyranosyl-(1→2)-4-azido-3-*O*-benzyl-4,6-dideoxy-α-d-mannopyranosyl-(1→2)-4-azido-3-*O*-benzyl-4,6-dideoxy-α-d-mannopyranosyl-(1→2)-4-azido-3-*O*-benzyl-4,6-dideoxy-α-d-mannopyranoside (16)**. A mixture of thioglycoside **9** (298 mg, 0.401 mmol), acceptor **12** (242 mg, 0.349 mmol) and mol. sieve 4 Å (550 mg) in CH_2_Cl_2_ (20 mL) was stirred at room temperature for 30 min, and then cooled to −35 °C. NIS (157 mg, 0.698 mmol) and a solution of TMSOTf (13 μL, 0.07 mmol) in CH_2_Cl_2_ (0.35 mL), and the resulting mixture was stirred for 1.5 h, while the temperature was gradually increased to −10 °C. The mixture was neutralized by adding pyridine (50 μL), diluted with CHCl_3_ (20 mL) and filtered through a Celite layer. The solids were washed with CHCl_3_ (3 × 10 mL), and the filtrate was washed with 0.5 M Na_2_S_2_O_3_ solution (20 mL) and water (20 mL). The organic solution was concentrated, and the residue was purified by column chromatography (toluene–EtOAc, 0→10%) to produce tetrasaccharide **16** (346 mg, 79%) as a colorless syrup, [α]_D_ +81.6 (с 1, CHCl_3_). ^1^H NMR (600 MHz, CDCl_3_): δ 7.46–7.26 (m, 20 H, Ar), 6.61 (br. s, 1 H, NH), 5.43 (dd, 1 H, *J*_2,1_ = 1.8 Hz, *J*_2,3_ = 3.1 Hz, H-2^D^), 4.95 (d, 1 H, *J*_1,2_ = 1.6 Hz, H-1^C^), 4.91 (d, 1 H, *J*_1,2_ = 1.6 Hz, H-1^B^), 4.87 (d, 1 H, *J*_1,2_ = 1.8 Hz, H-1d), 4.76–4.72 (m, 2 H, 2 benzylic H), 4.68–4.60 (m, 6 H, 5 benzylic H, H-1^A^), 4.56 (d, 1 H, *J* = 11.2 Hz, benzylic H) 3.90 (poorly resolved t, 1 H, H-2^C^), 3.85 (poorly resolved t, 1 H, H-2^B^), 3.82 (poorly resolved t, 1 H, H-2^A^), 3.80 (dd, 1 H, *J*_3,2_ = 3.2 Hz, *J*_3,4_ = 9.9 Hz, H-3^D^), 3.78–3.73 (OC*Ha*HbCH_2_CH_2_N), 3.71 (dd, 1 H, *J*_3,2_ = 2.9 Hz, *J*_3,4_ = 9.9 Hz, H-3^C^), 3.69 (dd, 1 H, *J*_3,2_ = 2.8 Hz, *J*_3,4_ = 9.9 Hz, H-3^B^), 3.66 (dd, 1 H, *J*_3,2_ = 3.1 Hz, *J*_3,4_ = 9.9 Hz, H-3^A^), 3.57–3.37 (m, 8 H, H-4^D^, H-5^A^, H-5^B^, H-5^C^, H-5^D^, OCHa*Hb*CH_2_CH_2_N, OCH_2_CH_2_C*H*_*2*_N), 3.35 (t, 1 H, *J* = 10.1 Hz, H-4^C^), 3.26 (t, 1 H, *J* = 9.9 Hz, H-4^B^), 3.24 (t, 1 H, *J* = 9.9 Hz, H-4^A^), 2.13 (s, 3 H, CH_3_CO), 1.90–1.84 (m, 2 H, OCH_2_C*H*_*2*_CH_2_N), 1.34–1.27 (m, 6 H, H-6^A^, H-6^B^), 1.23 (d, 3 H, *J*_6,5_ = 6.2 Hz, H-6^D^), 1.18 (d, 3 H, *J*_6,5_ = 6.1 Hz, H-6^C^). ^13^C NMR (101 MHz, CDCl_3_): δ 170.0 (CH_3_ *C*O) 157.2 (q, ^2^ *J*_C,F_ = 36.3 Hz, CF_3_ *C*O), 137.6, 137.4, 137.3, 129.2, 129.1, 128.8, 128.7, 128.6, 128.4, 128.3, 128.2 (Ar) 116.0 (q, ^1^ *J*_C,F_ = 288 Hz, *C*F_3_CO) 100.7 (C-1^B^), 100.3 (C-1^C^), 99.3 (C-1^D^), 99.1 (C-1^A^), 77.7 (C-3^A^), 76.9 (C-3^C^), 76.7 (C-3^B^), 75.6 (C-3^D^), 73.9 (C-2^A^), 73.7 (C-2^B^), 73.6 (C-2^C^), 72.4 (×2), 72.2, 71.7 (4 Ph*C*H_2_), 68.1 (C-5^B^), 68.0 (C-5^C^), 67.9 (C-5^D^), 67.6 (C-2^D^), 67.3 (C-5^A^), 66.0 (O*C*H_2_CH_2_CH_2_N), 64.4 (×2) (C-4^B^, C-4^A^), 64.2 (C-4^C^), 64.0 (C-4^D^), 38.2 (OCH_2_CH_2_ *C*H_2_N), 28.6 (OCH_2_ *C*H_2_CH_2_N), 21.2 (*C*H_3_CO), 18.8 (C-6^A^), 18.7 (C-6^B^), 18.6 (C-6^C^), 18.5 (C-6^D^). HRMS (ESI): calcd. for C_59_H_70_F_3_N_13_O_15_ [M + Na]^+^ *m/z* 1280.4959; found *m/z* 1280.4963.

**3-Trifluoroacetamidopropyl 4-azido-3-*O*-benzyl-4,6-dideoxy-α-d-mannopyranosyl-(1→2)-4-azido-3-*O*-benzyl-4,6-dideoxy-α-d-mannopyranosyl-(1→2)-4-azido-3-*O*-benzyl-4,6-dideoxy-α-d-mannopyranosyl-(1→2)-4-azido-3-*O*-benzyl-4,6-dideoxy-α-d-mannopyranoside (17)**. A solution of 2-acetate **16** (408 mg, 0.324 mmol) in MeOH (6.5 mL) was treated with methanolic sodium methoxide (65 μL) at room temperature for 5 h. The reaction mixture was worked up as described above for compound **15**. The title product **17** (324 mg, 82%) was isolated by column chromatography (toluene–EtOAc, 0→15%); a colorless foam, [α]_D_ +90.4 (с 1, CHCl_3_). ^1^H NMR (400 MHz, CDCl_3_): δ 7.43–7.28 (m, 20 H, Ar), 6.54 (br. s, 1 H, NH), 4.97 (d, 1 H, *J*_1,2_ = 1.6 Hz, H-1^D^), 4.93 (d, 1 H, *J*_1,2_ = 1.8 Hz, H-1^С^), 4.89 (d, 1 H, *J*_1,2_ = 1.8 Hz, H-1^B^), 4.76–4.57 (m, 9 H, H-1^A^, 4 PhC*H*_*2*_), 3.95 (poorly resolved t, 1 H, H-2^D^), 3.93 (poorly resolved t, 1 H, H-2^C^), 3.81 (poorly resolved t, 1 H, H-2^B^), 3.79 (poorly resolved t, 1 H, H-2^A^), 3.76–3.61 (m, 5 H, OC*Ha*HbCH_2_CH_2_N, H-3^A^, H-3^B^, H-3^C^, H-3^D^), 3.56–3.33 (m, 8H, H-4^D^, H-5^A^, H-5^B^, H-5^C^, H-5^D^, OCHa*Hb*CH_2_CH_2_N, OCH_2_CH_2_C*H*_*2*_N), 3.29 (t, 1 H, *J* = 10.0 Hz, H-4^C^), 3.24 (t, 1 H, *J* = 10.0 Hz, H-4^B^ 3.21 (t, 1 H, *J* = 10.0 Hz, H-4^A^), 2.29 (br. s, 1 H, OH) 1.88–1.81 (m, 2 H, OCH_2_C*H*_*2*_CH_2_N), 1.30–1.25 (m, 6 H, H-6^A^, H-6^B^), 1.20 (d, 3 H, *J*_6,5_ = 6.1 Hz, H-6^D^), 1.16 (d, 3 H, *J*_6,5_ = 6.1 Hz, H-6^C^). ^13^C NMR (101 MHz, CDCl_3_): δ 137.3, 137.1, 128.6, 128.4, 128.3, 128.2, 128.0 (Ar), 100.5 (×2) (C-1^B^, C-1^D^), 100.3 (C-1^C^), 98.9 (C-1^A^), 77.7 (C-3^D^), 77.5 (C-3^A^), 76.9 (C-3^B^), 76.5 (C-3^C^), 73.7 (C-2^A^), 73.6 (C-2^B^), 73.3 (C-2^C^), 72.19 (×2), 72.16, 72.1 (4 Ph*C*H_2_), 67.91 (C-5^B^), 67.85 (C-5^С^), 67.5 (C-5^A^), 67.4 (C-5^D^), 67.2 (C-2^D^), 65.8 (O*C*H_2_CH_2_CH_2_N), 64.2 (×3) (C-4^A^, C-4^B^, C-4^D^), 63.9 (C-4^C^), 38.0 (OCH_2_CH_2_ *C*H_2_N), 28.4 (OCH_2_ *C*H_2_CH_2_N), 18.6 (×2) (C-6^A^, C-6^B^) 18.5 (C-6^D^), 18.3 (C-6^C^). HRMS (ESI): calcd. for C_57_H_68_F_3_N_13_O_14_ [M + Na]^+^ *m/z* 1238.4853; found *m/z* 1238.4848.

**3-Trifluoroacetamidopropyl 2-*O*-acetyl-4-azido-3-*O*-benzyl-4,6-dideoxy-α-d-mannopyranosyl-(1→2)-4-azido-3-*O*-benzyl-4,6-dideoxy-α-d-mannopyranosyl-(1→2)-4-azido-3-*O*-benzyl-4,6-dideoxy-α-d-mannopyranosyl-(1→2)-4-azido-3-*O*-benzyl-4,6-dideoxy-α-d-mannopyranosyl-(1→2)-4-azido-3-*O*-benzyl-4,6-dideoxy-α-d-mannopyranoside (18)**. A mixture of donor **6** (58 mg, 0.118 mmol), acceptor **17** (120 mg, 0.099 mmol) and mol sieve 4 Å (200 mg) in CH_2_Cl_2_ (9 mL) was stirred at room temperature for 30 min, and then cooled to −40 °C. NIS (45 mg, 0.198 mmol) and a solution of TMSOTf (4 μL, 0.02 mmol) in CH_2_Cl_2_ (24 μL), and the mixture was stirred for 10 min, gradually increasing the temperature to −30 °C. The mixture was neutralized by adding pyridine (20 μL), diluted with CHCl_3_ (10 mL), and filtered through a Celite layer. The solids were washed with CHCl_3_ (3 × 5 mL), and the filtrate was washed with 0.5 M Na_2_S_2_O_3_ solution (10 mL) and water (20 mL), and concentrated. Column chromatography of the residue (toluene–EtOAc, 0→10%) yielded pentasaccharide **18** (134 mg, 89%) as a colorless foam, [α]_D_ +92.3 (с 1, CHCl_3_). ^1^H NMR (400 MHz, CDCl_3_): δ 7.41–7.28 (m, 25 H, Ar), 6.52 (br. s, 1 H, NH), 5.40 (poorly resolved t, 1 H, H-2^E^), 4.95 (d, 1 H, *J*_1,2_ = 1.5 Hz, H-1^D^), 4.88–4.83 (m, 3 H, H-1^B^, H-1^E^, H-1^C^), 4.74–4.69 (m, 2 H, 2 benzylic H), 4.67–4.51 (m, 9 H, H-1^A^, 8 benzylic H), 3.87 (poorly resolved t, 1 H, H-2^D^), 3.83 (poorly resolved t, 1 H, H-2^C^), 3.80–3.60 (m, 8 H, H-2^A^, H-2^B^, H-3^A^, H-3^B^, H-3^C^, H-3^E^, H-3^D^, OC*Ha*HbCH_2_CH_2_N), 3.56–3.30 (m, 10 H, H-4^D^, H-4^E^, H-5^A^, H-5^B^, H-5^C^, H-5^D^, H-5^E^, OCHa*Hb*CH_2_CH_2_N, OCH_2_CH_2_C*H*_*2*_N), 3.24–3.16 (m, 3 H, H-4^A^, H-4^B^, H-4^C^), 2.10 (s, 3 H, CH_3_CO), 1.88–1.80 (m, 2 H, OCH_2_C*H*_*2*_CH_2_N), 1.27 (d, 3 H, *J*_6,5_ = 6.2 Hz, H-6^A^), 1.25 (d, 3 H, *J*_6,5_ = 6.4 Hz, H-6^D^), 1.21 (d, 3 H, *J*_6,5_ = 6.2 Hz, H-6^E^), 1.18 (d, 3 H, *J*_6,5_ = 6.0 Hz, H-6^B^), 1.14 (d, 3 H, *J*_6,5_ = 6.0 Hz, H-6^C^). ^13^C NMR (101 MHz, CDCl_3_): δ 169.8 (CH_3_ *C*O), 128.6, 128.4, 128.3, 128.1, 128.0 (Ar), 100.5 (C-1^B^), 100.2 (C-1^D^), 100.1 (C-1^E^), 99.1 (C-1^С^), 98.9 (C-1^A^), 77.5 (C-3^A^), 76.8 (C-3^B^), 76.6 (C-3^D^), 76.4 (C-3^C^), 75.4 (C-3^E^), 73.8 (C-2^A^), 73.6 (C-2^B^), 73.5 (C-2^D^), 73.4 (C-2^C^), 72.2 (×3), 72.0, 71.6 (5 Ph*C*H_2_), 67.9 (×3) (C-5^B^, C-5^D^, C-5^E^), 67.7 (C-5^C^), 67.5 (C-5^A^), 67.2 (C-2^E^), 65.8 (O*C*H_2_CH_2_CH_2_N), 64.2 (×3) (C-4^A^, C-4^A^, C-4^C^), 64.1 (C-4^D^), 63.9 (C-4^E^), 38.0 (OCH_2_CH_2_ *C*H_2_N), 28.4 (OCH_2_ *C*H_2_CH_2_N), 21.0 (*C*H_3_CO), 18.6 (×2), 18.5 (×2), 18.4 (C-6^A^, C-6^B^, C-6^C^, C-6^D^, C-6^E^). HRMS (ESI): calcd. for C_72_H_85_F_3_N_16_O_18_ [M + Na]^+^ *m/z* 1541.6065; found *m/z* 1541.6072.

**3-Trifluoroacetamidopropyl 4-azido-3-*O*-benzyl-4,6-dideoxy-α-d-mannopyranosyl-(1→2)-4-azido-3-*O*-benzyl-4,6-dideoxy-α-d-mannopyranosyl-(1→2)-4-azido-3-*O*-benzyl-4,6-dideoxy-α-d-mannopyranosyl-(1→2)-4-azido-3-*O*-benzyl-4,6-dideoxy-α-d-mannopyranosyl-(1→2)-4-azido-3-*O*-benzyl-4,6-dideoxy-α-d-mannopyranoside (19)**. 1 M sodium methoxide (20 μL) was added to a solution of 2-acetate **18** (162 mg, 0.107 mmol) in MeOH (2.2 mL), the mixture was stirred for 4.5 h at room temperature, and then worked up as described above for compound **15**. Column chromatography (toluene–EtOAc, 0→15%) produced title product **19** (122 mg, 77%) as a colorless foam, [α]_D_ +98.7 (с 1, CHCl_3_). ^1^H NMR (600 MHz, CDCl_3_): δ 7.42–7.29 (m, 25 H, Ar), 6.54 (br. s, 1 H, NH), 4.97 (d, 1 H, *J*_1,2_ = 1.7 Hz, H-1^B^), 4.96 (d, 1 H, *J*_1,2_ = 1.8 Hz, H-1^C^), 4.86 (d, 1 H, *J*_1,2_ = 1.7 Hz, H-1^E^), 4.85 (d, 1 H, *J*_1,2_ = 1.7 Hz, H-1^D^), 4.74–4.57 (m, 11 H, H-1^A^, 10 benzylic H), 3.99 (br. s, 1 H, H-2^E^), 3.94–3.93 (poorly resolved t, 1 H, H-2^C^), 3.82 ((poorly resolved t, 1 H, H-2^D^), 3.79–3.77 (m, 2 H, H-2^A^, H-2^B^), 3.75–3.69 (m, 3 H, H-3^B^, H-3^C^, OC*Ha*HbCH_2_CH_2_N), 3.66–3.61 (m, 3 H, H-3^A^, H-3^D^, H-3^E^), 3.55–3.34 (m, 9 H, H-4c, H-5a, H-5b, H-5c, H-5e, H-5d, OCHa*Hb*CH_2_CH_2_N, OCH_2_CH_2_C*H*_*2*_N), 3.31 (t, 1 H, *J* = 10.1 Hz, H-4^B^) 3.24–3.17 (m, 3 H, H-4^A^, H-4^D^, H-4^E^), 2.29 (br. s, 1 H, OH), 1.87–1.81 (m, 2 H, OCH_2_C*H*_*2*_CH_2_N), 1.27 (d, 3 H, *J*_6,5_ = 6.2 Hz, H-6), 1.25 (d, 3 H, *J*_6,5_ = 6.2 Hz, H-6), 1.20 (d, 3 H, *J*_6,5_ = 6.2 Hz, H-6), 1.18 (d, 3 H, *J*_6,5_ = 6.2 Hz, H-6), 1.14 (d, 3 H, *J*_6,5_ = 6.2 Hz, H-6). ^13^C NMR (151 MHz, CDCl_3_): δ 128.7, 128.6, 128.4, 128.4, 128.3, 128.3, 128.1 (Ar), 100.5 (×2) (C-1^B^, C-1^C^), 100.2 (×2) (C-1^D^, C-1^E^), 98.9 (C-1^A^), 77.7 (C-3^E^), 77.5 (C-3^C^), 77.0 (C-3^B^), 76.6 (C-3^D^), 76.4 (C-3^A^), 73.7 (C-2^E^), 73.6 (C-2^A^), 73.4 (C-2^D^), 73.3 (C-2^C^), 72.2 (×4) 72.1 (5 Ph*C*H_2_), 67.9 (×2) 67.8 67.5, 67.4, (C-5^A^, C-5^B^, C-5^C^, C-5^D^, C-5^E^), 67.2 (C-2^E^), 65.8 (O*C*H_2_CH_2_CH_2_N), 64.2 (×3), 64.1, 63.8 (5 C-4), 38.0 (OCH_2_CH_2_ *C*H_2_N), 28.4 (OCH_2_ *C*H_2_CH_2_N), 18.6 (×2),18.5 (×2), 18.3 (5 C-6). HRMS (ESI): calcd. for C_70_H_83_F_3_N_16_O_17_ [M + Na]^+^ *m/z* 1499.5965; found *m/z* 1499.5966.

**3-Trifluoroacetamidopropyl 4-amino-3-*O*-benzyl-4,6-dideoxy-α-d-mannopyranosyl-(1→2)-4-amino-3-*O*-benzyl-4,6-dideoxy-α-d-mannopyranoside (20)**. A mixture of diazide **11α** (85 mg, 0.123 mmol) and Pd(OH)_2_/C (31 mg) in MeOH (3 mL) was vigorously stirred at 35 °C under hydrogen for 30 min. Then the catalyst was filtered off through a Celite layer, thoroughly washed with MeOH (4 × 5 mL), and the filtrate was concentrated. Column chromatography of the residue (CH_2_Cl_2_ – MeOH, 0→15%) yielded diamine **20** (62 mg, 78%) as a colorless syrup. ^1^H NMR (600 MHz, CD_3_OD): δ 7.44–7.27 (m, 10 H, Ar), 4.91 (d, 1 H, *J*_1,2_ = 1.9 Hz, H-1^B^), 4.79 (d, 1 H, *J*_1,2_ = 1.9 Hz, H-1^A^), 4.72 (d, 1 H, *J* = 11.7 Hz, PhC*Ha*Hb), 4.68 (d, 1 H, *J* = 11.4 Hz, PhC*Ha*Hb′), 4.58 (d, 1 H, *J* = 11.4 Hz, PhCHa*Hb*′), 4.51 (d, 1 H, *J* = 11.7 Hz, PhCHa*Hb*), 4.08 (poorly resolved t, 1 H, H-2^B^), 3.95 (poorly resolved t, 1 H, H-2^A^), 3.78 (dq, 1 H, *J*_5,6_ = 6.2 Hz, *J*_5,4_ = 10.1 Hz, H-5^B^), 3.71–3.66 (m, 2 H, H-3^A^, OC*Ha*HbCH_2_CH_2_N), 3.65–3.59 (m, 2 H, H-5^A^, H-3^B^), 3.42–3.37 (m, 3 H, OCHa*Hb*CH_2_CH_2_N, OCH_2_CH_2_C*H*_*2*_N), 3.02 (t, 1 H, *J* = 10.1 Hz, H-4^B^), 2.85 (t, 1 H, *J* = 9.9 Hz, H-4^A^), 1.87–1.81 (m, 2 H, OCH_2_C*H*_*2*_CH_2_N), 1.25 (d, 3 H, *J*_6,5_ = 6.2 Hz, H-6^B^), 1.23 (d, 3 H, *J*_6,5_ = 6.2 Hz, H-6^A^). ^13^C NMR (151 MHz, CD_3_OD): δ 139.2, 139.0, 129.9, 129.8, 129.7, 129.7, 129.3, 129.2 (Ar), 103.4 (C-1^B^), 100.5 (C-1^A^), 78.9 (C-3^A^), 77.0 (C-3^B^), 74.6 (C-2^A^), 72.7, 71.3 (2 Ph*C*H_2_), 69.3 (C-5^A^), 69.2 (C-5^B^), 66.6 (C-2^B^), 65.9 (O*C*H_2_CH_2_CH_2_N), 54.9 (C-4^A^), 54.2 (C-4^B^), 38.0 (OCH_2_CH_2_ *C*H_2_N), 29.9 (OCH_2_ *C*H_2_CH_2_N), 18.5 (C-6^A^), 18.3 (C-6^B^). HRMS (ESI): calcd. for C_31_H_42_F_3_N_3_O_8_ [M + Na]^+^ *m/z* 664.2817; found *m/z* 664.2816.

**3-Trifluoroacetamidopropyl 3-*O*-benzyl-4,6-dideoxy-4-formamido-α-d-mannopyranosyl-(1→2)-3-*O*-benzyl-4,6-dideoxy-4-formamido-α-d-mannopyranoside (21)**. Formic acid (22 μL, 0.57 mmol) and DCC (59 mg, 0.285 mmol) were added to a solution of diamine **20** (61 mg, 0.095 mmol) in CH_2_Cl_2_ and MeOH (9:1, 3 mL), and the mixture was stirred at room temperature for 1.5 h. Then more formic acid (5 μL, 0.130 mmol) and DCC (16 mg, 0.077 mmol) were added, and stirring was continued for the next 1.5 h. The solvents were evaporated, the residue was suspended in CH_2_Cl_2_ (3 mL), and the precipitate of dicyclohexylurea was filtered off and washed with CH_2_Cl_2_ (3 × 2 mL). The filtrate was concentrated to a volume of ∼2 mL, and the resulting solution was subjected to column chromatography (CH_2_Cl_2_ – MeOH, 0→10%) to produce bis(formamide) **21** (57 mg, 87%) as a colorless amorphous solid. HRMS (ESI): calcd. for C_33_H_42_F_3_N_3_O_10_ [M + Na]^+^ *m/z* 720.2709; found *m/z* 720.2715.

**3-Trifluoroacetamidopropyl 4,6-dideoxy-4-formamido-α-d-mannopyranosyl-(1→2)-4,6-dideoxy-4-formamido-α-d-mannopyranoside (22)**. Pd(OH)_2_/C (20 mg) was added to a solution of disaccharide **21**(57 mg, 0.082 mmol) in MeOH (3 mL) and the mixture was vigorously stirred under hydrogen at room temperature for 24 h. More catalyst (7 mg) was added and stirring was continued for the next 1.5 h. The catalyst was filtered of through a Celite layer, washed with MeOH (4 × 4 mL), and the filtrate was concentrated. The residue was purified by column chromatography (CH_2_Cl_2_ – MeOH, 15→25%) to give debenzylated product **22** (34 mg, 94%) as a colorless amorphous solid. HRMS (ESI): calcd. for C_19_H_30_F_3_N_3_O_10_ [M + H]^+^ *m/z* 518.1953; found *m/z* 518.1956.

**3-Aminopropyl 4,6-dideoxy-4-formamido-α-d-mannopyranosyl-(1→2)-4,6-dideoxy-4-formamido-α-d-mannopyranoside (1a)**. Ambersep 900 (OH^−^) (2 mL) was added to a solution of disaccharide **22** (33 mg, 0.064 mmol) in 50% aqueous MeOH (3 mL). The mixture was kept for 1 h with periodic shaking, and then the resin was filtered off and washed with 50% aqueous MeOH (6 × 3 mL). The filtrate was concentrated, and the residue was subjected to gel chromatography to afford 3-aminopropyl glycoside **1a** (25 mg, 85%) as a white amorphous solid; contained a non-stoichiometric amount (∼0.40 equiv.) of AcOH; [α]_D_ +31.0 (с 0.25, water). ^1^H NMR (600 MHz, D_2_O): δ 8.20, 8.19 (2 s, 1.6 H, *H*_*Z*_C(O)NH), 8.03, 8.02 (2 s, 0.4 H, *H*_*E*_C(O)NH), 5.03 (br. s, 0.2 H, H_*E*_-1^B^) 5.02 (d, 0.8 H, *J*_1,2_ = 1.7 Hz, H_*Z*_-1^B^), 4.94 (br. s, 0.8 H, H_*Z*_-1^A^), 4.92 (br. s, 0.2 H, H_*E*_-1^A^), 4.12–4.0 (m, 1 H, H-2^B^), 4.02–3.77 (m, 7.6 H, H-2^A^, H-3^A^, H-3^B^, H_*Z*_-4^A^, H_*Z*_-4^B^, H-5^A^, H-5^B^, OC*Ha*HbCH_2_CH_2_N), 3.60–3.55 (m, 1 H, OCHa*Hb*CH_2_CH_2_N), 3.40 (t, 0.2 H, *J* = 10.3 Hz, H_*E*_-4), 3.38 (t, 0.2 H, H_*E*_-4), 3.16–3.07 (m, 2 H, OCH_2_CH_2_C*H*_*2*_N), 2.02–1.95 (m, 2 H, OCH_2_C*H*_*2*_CH_2_N), 1.90 (s, 1.2 H, CH_3_COO^−^), 1.28–1.20 (m, 6 H, H-6^A^, H-6^B^). ^13^C NMR (151 MHz, D_2_O): δ 167.9, 167.8 (H_*E*_ *C*ONH), 164.92 (H_*Z*_ *C*ONH), 102.2 (C_*E*_-1^B^), 102.1 (C_*Z*_-1^B^), 98.4 (C-1^A^), 78.0 (C_*Z*_-2^A^), 77.9 (C_*E*_-2^A^), 69.8 (H-2^B^), 68.0, 67.9, 67.6, 67.5 (С-3^A^, C-3^B^, C-5^A^, C-5^B^), 65.2 (O*C*H_2_CH_2_CH_2_N), 56.9, 56.7 (C_*E*_-4^A^, C_*E*_-4^B^), 52.1, 51.9, 51.7 (C_*Z*_-4^A^, C_*Z*_-4^B^), 37.4 (OCH_2_CH_2_ *C*H_2_N), 26.7 (OCH_2_ *C*H_2_CH_2_N), 16.9, 16.7 (C-6^A^, C-6^B^). HRMS (ESI): calcd. for C_17_H_32_N_3_O_9_ [M + Na]^+^ *m/z* 444.1952; found *m/z* 444.1953.

**3-Aminopropyl 4,6-dideoxy-4-formamido-α-d-mannopyranosyl-(1→2)-4,6-dideoxy-4-formamido-α-d-mannopyranosyl-(1→2)-4,6-dideoxy-4-formamido-α-d-mannopyranoside (2a)**. Compound **2a** was synthesized starting from trisaccharide **15** (103 mg, 0.108 mmol) according to the procedure used for transformation of disaccharide **12** into target compound **1a** to give 39.8 mg (66%) of trisaccharide **2a** as a white fluffy solid; contained a non-stoichiometric amount (∼0.40 equiv.) of AcOH, [α]_D_ +36.5 (с 1, water). ^1^H NMR (600 MHz, D_2_O): δ 8.21–8.18 (m, 2.4 H, H_*Z*_CON), 8.04–8.02 (m, 0.6 H, H_*E*_CON), 5.21–4.87 (m, 3 H, 3 H-1), 4.18–3.78 (m, 12.4 H, 3 H-2, 3 H-3, 3 H-4_*Z*_, 3 H-5, OC*Ha*HbCH_2_CH_2_N), 3.60–3.55 (m, 1 H, OCHa*Hb*CH_2_CH_2_N), 3.45–3.35 (m, 0.6 H, 3 H-4_*E*_), 3.16–3.07 (m, 2 H, OCH_2_CH_2_C*H*_*2*_N), 2.01–1.95 (m, 2 H, OCH_2_C*H*_*2*_CH_2_N), 1.29–1.18 (m, 9 H, 3 H-6). ^13^C NMR (151 MHz, D_2_O): δ 169.0, 168.9 (H_*E*_CON), 166.0 (H_*Z*_CON), 103.2, 103.1, 103.0, 101.8, 99.6 (3 C-1), 78.8, 78.7, 78.5, 78.4 (C-2^A^, C-2^B^), 70.1, 69.4, 69.3, 69.1, 69.0, 68.8, 68.6, 68.5, 68.4, 68.3 (C-3^A^, C-5^A^, C-3^B^, C-5^B^, C-2^C^, C-3^C^, C-5^C^), 66.3 (O*C*H_2_CH_2_CH_2_N), 58.0, 57.8 (3 C-4_*E*_), 53.2, 53.1, 53.0, 52.9 (3 C-4_*Z*_), 38.5 (OCH_2_CH_2_ *C*H_2_N), 27.8 (OCH_2_ *C*H_2_CH_2_N), 24.4 (*C*H_3_COO^−^), 18.1, 18.0, 17.9, 17.8 (3 C-6). HRMS (ESI): calcd. for C_24_H_42_F_3_N_4_O_13_ [M + Na]^+^ *m/z* 617.2646; found *m/z* 617.2641.

**3-Aminopropyl 4,6-dideoxy-4-formamido-α-d-mannopyranosyl-(1→2)-4,6-dideoxy-4-formamido-α-d-mannopyranosyl-(1→2)-4,6-dideoxy-4-formamido-α-d-mannopyranosyl-(1→2)-4,6-dideoxy-4-formamido-α-d-mannopyranoside (3a)**. Compound **3a** was synthesized starting from tetrasaccharide **17** (85 mg, 0.071 mmol) according to the procedure used for transformation of disaccharide **12** into target compound **1a** to give 27.7 mg (53%) of tetrasaccharide **3a** as a white fluffy solid; contained a non-stoichiometric amount (∼0.35 equiv.) of AcOH, [α]_D_ +39.4 (с 1, water). ^1^H NMR (600 MHz, D_2_O): δ 8.22–8.18 (m, 3.2 H, H_*Z*_CON), 8.05–8.01 (m, 0.8 H, H_*E*_CON), 5.23–4.87 (m, 4 H, 4 H-1), 4.19–3.78 (m, 16.2 H, 4 H-2, 4 H-3, 4 H-4_*Z*_, OC*Ha*HbCH_2_CH_2_N), 3.61–3.55 (m, 1 H, OCHa*Hb*CH_2_CH_2_N), 3.46–3.35 (m, 0.8 H, 4 H-4_*E*_), 3.16–3.07 (m, 2 H, OCH_2_CH_2_C*H*_*2*_N), 2.02–1.94 (m, 2 H, OCH_2_C*H*_*2*_CH_2_N), 1.90 (s, 1.1 H, CH_3_COO^−^), 1.29–1.17 (m, 12 H, 4 H-6). ^13^C NMR (151 MHz, D_2_O): δ 168.9 (H_*E*_CON), 166.0 (H_*Z*_CON), 103.1, 103.0, 101.7, 101.6, 99.5 (4 C-1), 78.7, 78.4, 78.3, 78.1 (C-2^A^, C-2^B^, C-2^C^), 70.0, 69.4, 69.3, 69.1, 69.0, 68.7, 68.5, 68.2 (4 C-3, 4 C-5, C-2^D^), 66.2 (O*C*H_2_CH_2_CH_2_N), 58.0, 57.8 (C-4_*E*_), 53.2, 53.1, 53.0, 52.8 (C-4_*Z*_), 38.5 (OCH_2_CH_2_ *C*H_2_N), 27.8 (OCH_2_ *C*H_2_CH_2_N), 24.4 (*C*H_3_COO^−^), 18.0, 17.9, 17.8 (4 C-6). HRMS (ESI): calcd. for C_31_H_53_N_5_O_17_ [M + Na]^+^ *m/z* 790.3327; found *m/z* 790.3329.

**3-Aminopropyl 4,6-dideoxy-4-formamido-α-d-mannopyranosyl-(1→2)-4,6-dideoxy-4-formamido-α-d-mannopyranosyl-(1→2)-4,6-dideoxy-4-formamido-α-d-mannopyranosyl-(1→2)-4,6-dideoxy-4-formamido-α-d-mannopyranosyl-(1→2)-4,6-dideoxy-4-formamido-α-d-mannopyranoside (4a)**. Compound **4a** was synthesized starting from pentaasaccharide **19** (47 mg, 0.032 mmol) according to the procedure used for transformation of disaccharide **12** into target compound **1a** to give 10.0 mg (39%) of pentaasaccharide **4a** as a white fluffy solid; contained a non-stoichiometric amount (∼0.30 equiv.) of AcOH, [α]_D_ +42.5 (с 0.5, water). ^1^H NMR (600 MHz, D_2_O): δ 8.18 (br s, 1.5 H, H_*Z*_CON), 8.03, 8.01 (2 s, 0.7 H, H_*E*_CON), 5.21–4.85 (m, 5 H, 5 H-1), 4.19–3.76 (m, 17.3 H, 5 H-2, 5 H-3, 5 H-4_*Z*_, OC*Ha*HbCH_2_CH_2_N), 3.60–3.53 (m, 1 H, OCHa*Hb*CH_2_CH_2_N), 3.45–3.33 (m, 0.8 H, 5 H-4_*E*_), 3.16–3.05 (m, 2 H, OCH_2_CH_2_C*H*_*2*_N), 2.01–1.93 (m, 2 H, OCH_2_C*H*_*2*_CH_2_N), 1.89 (s, 1.2 H, CH_3_COO^−^), 1.33–1.15 (m, 15 H, 5 H-6). ^13^C NMR (151 MHz, D_2_O) δ 167.9 (H_*E*_CON), 165.0 (H_*Z*_CON), 102.0, 100.7, 100.6, 98.5 (5 C-1), 77.6, 77.2, 77.0 (C-2^A^, C-2^B^, C-2^C^, C-2^D^), 69.0, 68.3, 68.0, 67.9, 67.7, 67.5 (5 C-3, 5 C-5, C-2^E^), 65.2 (O*C*H_2_CH_2_CH_2_N), 56.9 (C-4_*E*_), 52.2, 52.0, 51.7 (C-4_*Z*_), 37.4 (OCH_2_CH_2_ *C*H_2_N), 26.8 (OCH_2_ *C*H_2_CH_2_N), 17.0, 16.9, 16.8, 16.7 (5 C-6). HRMS (ESI): calcd. for C_38_H_64_N_6_O_21_ [M + H]^+^ *m/z* 941.4190; found *m/z* 941.4197.

**Preparation of fluorescein labelled glycoconjugates 1b-4b**. General procedure: FITC was conjugated to synthetic oligosaccharides **1a**–**4a** as described previously (Mukhametova et al., 2024b). Briefly, to aminopropyl glycoside **1a**–**4a** (1 equiv.) and Na_2_CO_3_ (3 equiv.) water solution fluorescein isothiocyanate (FITC) (1.2 eq.) in DMF was added. The obtained mixture was vigorously mixed and kept at 60 °C for 2 h. The reaction mixture was concentrated *in vacuo*, dissolved in water (300 μL) and the mixture was loaded onto a Sep-Pak C-18 cartridge, which was preliminarily washed with methanol and then with excess water. The preliminarily washed cartridge was eluted with 2 mL portions of methanol–water mixture (from 0 to 60 vol% of methanol) with the concentration increasing in increments of 5 vol%. Product **1b** was collected at eluent concentrations between 10 and 20 vol%, products **2b** between 25 and 40 vol%, **3b** between 30 and 40 vol%, **4b** between 15 and 30 vol%. After evaporation and lyophilization, the products **1b**–**4b** were obtained as light orange fluffy solids. The purity of the products was confirmed by thin-layer chromatography.

**Synthesis of disaccharide tracer 1b**. The fluorescent labelling of aminoethyl glycoside **1a** (1.07 mg, 2.54 μmol) as described in general procedure, gave a light orange product (1.81 mg, 88%). HRMS (ESI) calcd. for C_38_H_42_N_4_O_14_S [M + Na]^+^ 833.2310 was found to be 833.2306.

**Synthesis of trisaccharide tracer 2b**. The fluorescent labelling of aminoethyl glycoside **2a** (1.00 mg, 1.69 μmol) as described in general procedure, gave a light orange product (1.31 mg, 80%). HRMS (ESI) calcd. for calcd. for **3b** C_45_H_53_N_5_O_18_S [M + Na]^+^ 1006.2999 found 1006.2987.

**Synthesis of tetrasaccharide tracer 3b**. The fluorescent labelling of aminoethyl glycoside **3a** (1.54 mg, 2.00 μmol) as described in general procedure, gave a light orange product (1.57 mg, 83%). HRMS (ESI) calcd. for C_52_H_64_N_6_O_22_S [M + Na]^+^ 1179.3687 was found to be 1179.3683.

**Synthesis of pentasaccharide tracer 4b**. The fluorescent labelling of aminoethyl glycoside **4a** (0.68 mg, 0.723 μmol) as described in general procedure, gave a light orange product (0.8234 mg, 86%). HRMS (ESI) calcd. for C_59_H_75_N_7_O_26_S [M + Na]^+^ 1352.4375 was found to be 1352.4373.

### Serum samples

Positive serum samples from multiple brucellosis-unfavorable farms (N = 19) were provided by Federal state budgetary institution «The Russian state center for animal feed and drug standardization and quality» (Moscow, Russia). These *Brucella*-positive sera were confirmed by at least two serological assays, including RBT, CFT, and ELISA. Brucellosis negative (N = 20) samples were provided from brucellosis-free farms and the reaction to all serological tests for brucellosis was negative.

### Fluorescence polarization assay

FITC-labeled glycoconjugates **23**, **1b**–**4b** tracer working solutions (2.5 nM) in 10 mM phosphate buffer with 0.15 M NaCl, pH 7.4, were prepared so that the fluorescence intensity of the solutions was 10 times more than the buffer background signal, or roughly 200,000 U. The tracer working solution (1.0 mL) was then mixed with 10 µL of the tested serum, then the tube was vigorously shaken and after 1–2 min incubation time at 20°C. The intensity and polarization of fluorescence were measured using a portable device Sentry-200 (Ellie LLC, Germantown, WI, USA) λex = 485 nm and λem = 535 nm). Every measurement was carried out three times. Analysis and statistical evaluation of the experimental data was performed using SigmaPlot 11 (Systat Software Inc., Palo Alto, CA, United States) software.

## Data Availability

The original contributions presented in the study are included in the article/Supplementary Material, further inquiries can be directed to the corresponding author.
