# Supplementary material for: Synthesis of oligo-α-(1→2)-4,6-dideoxy-4-formamido-d-mannopyranosides related to the A epitope of the Brucella O-polysaccharide and their use for assaying of serum immunoglobulins
Source: Front Chem. 2025 Aug 29;13:1662885. doi: 10.3389/fchem.2025.1662885 (PMC12426761; doi:10.3389/fchem.2025.1662885)

Synthesis of oligo-α-(1→2)-4,6-dideoxy-4-formamido-D-mannopyranosides related to the A epitope of the *Brucella* O-polysaccharide and their use for assaying of serum immunoglobulins

Timur M. Volkov,[1] Yury E. Tsvetkov,[1] Dmitry V. Yashunsky,[1] Anton N. Kuznetsov,[1] Oleg D. Sclyarov,[2] Olesia V. Babicheva [2], Dmitry O. Zherdev,[3] Liliya I. Mukhametova,[3] Sergei A. Eremin,[3] Vadim B. Krylov,[1] Nikolay E. Nifantiev*[1]

[1]Laboratory of Glycoconjugate Chemistry, N. D. Zelinsky Institute of Organic Chemistry, Russian Academy of Sciences, 47 Leninsky prosp., 119991 Moscow, Russia.

[2]Russian State Centre of Quality and Standardization of Veterinary Drugs and Feeds, 123022 Moscow, Russia.

[3]Department of Chemistry, Moscow State University, 119991 Moscow, Russia

*** Correspondence:**
Nikolay E. Nifantiev *nen@ioc.ac.ru*

Supplementary Material

Supplementary Data

^{1}H and ^{13}C NMR spectra of synthesized compounds

3-Aminopropyl 4,6-dideoxy-4-formamido-α-D-mannopyranosyl-(1→2)-4,6-dideoxy-4-formamido-α-D-mannopyranoside (**1a**) 3

3-Aminopropyl 4,6-dideoxy-4-formamido-α-D-mannopyranosyl-(1→2)-4,6-dideoxy-4-formamido-α-D-mannopyranosyl-(1→2)-4,6-dideoxy-4-formamido-α-D-mannopyranoside (**2a**) 4

3-Aminopropyl 4,6-dideoxy-4-formamido-α-D-mannopyranosyl-(1→2)-4,6-dideoxy-4-formamido-α-D-mannopyranosyl-(1→2)-4,6-dideoxy-4-formamido-α-D-mannopyranosyl-(1→2)-4,6-dideoxy-4-formamido-α-D-mannopyranoside (**3a**) 5

3-Aminopropyl 4,6-dideoxy-4-formamido-α-D-mannopyranosyl-(1→2)-4,6-dideoxy-4-formamido-α-D-mannopyranosyl-(1→2)-4,6-dideoxy-4-formamido-α-D-mannopyranosyl-(1→2)-4,6-dideoxy-4-formamido-α-D-mannopyranosyl-(1→2)-4,6-dideoxy-4-formamido-α-D-mannopyranoside (**4a**) 6

5-*tert*-Butyl-2-methylphenyl 2-O-acetyl-4-azido-3-O-benzyl-4,6-dideoxy-1-thio-α-D-mannopyranoside (**6**) 7

5-*tert*-Butyl-2-methylphenyl 4-azido-3-O-benzyl-4,6-dideoxy-1-thio-α-D-mannopyranoside (**7**) 8

5-*tert*-Butyl-2-methylphenyl 2-O-acetyl-4-azido-3-O-benzyl-4,6-dideoxy-α-D-mannopyranosyl-(1→2)-4-azido-3-O-benzyl-4,6-dideoxy-1-thio-α-D-mannopyranoside (**9**) 9

3-Trifluoroacetamidopropyl 2-O-acetyl-4-azido-3-O-benzyl-4,6-dideoxy-α-D-mannopyranosyl-(1→2)-4-azido-3-O-benzyl-4,6-dideoxy-α-D-mannopyranoside (**11α**) 10

Trifluoroacetamidopropyl 2-O-acetyl-4-azido-3-O-benzyl-4,6-dideoxy-α-D-mannopyranosyl-(1→2)-4-azido-3-O-benzyl-4,6-dideoxy-β-D-mannopyranoside (**11β**) 11

3-Trifluoroacetamidopropyl 4-azido-3-O-benzyl-4,6-dideoxy-α-D-mannopyranosyl-(1→2)-4-azido-3-O-benzyl-4,6-dideoxy-α-D-mannopyranoside (**12**) 12

Trifluoroacetamidopropyl 2-O-acetyl-4-azido-3-O-benzyl-4,6-dideoxy-α-D-mannopyranosyl-(1→2)-4-azido-3-O-benzyl-4,6-dideoxy-α-D-mannopyranosyl-(1→2)-4-azido-3-O-benzyl-4,6-dideoxy-α-D-mannopyranoside (**14**) 13

Trifluoroacetamidopropyl 4-azido-3-O-benzyl-4,6-dideoxy-α-D-mannopyranosyl-(1→2)-4-azido-3-O-benzyl-4,6-dideoxy-α-D-mannopyranosyl-(1→2)-4-azido-3-O-benzyl-4,6-dideoxy-α-D-mannopyranoside (**15**) 14

Trifluoroacetamidopropyl 2-O-acetyl-4-azido-3-O-benzyl-4,6-dideoxy-α-D-mannopyranosyl-(1→2)-4-azido-3-O-benzyl-4,6-dideoxy-α-D-mannopyranosyl-(1→2)-4-azido-3-O-benzyl-4,6-dideoxy-α-D-mannopyranosyl-(1→2)-4-azido-3-O-benzyl-4,6-dideoxy-α-D-mannopyranoside (**16**)................................ 15

Trifluoroacetamidopropyl 4-azido-3-O-benzyl-4,6-dideoxy-α-D-mannopyranosyl-(1→2)-4-azido-3-O-benzyl-4,6-dideoxy-α-D-mannopyranosyl-(1→2)-4-azido-3-O-benzyl-4,6-dideoxy-α-D-mannopyranosyl-(1→2)-4-azido-3-O-benzyl-4,6-dideoxy-α-D-mannopyranoside (**17**)................................ 16

Trifluoroacetamidopropyl 2-O-acetyl-4-azido-3-O-benzyl-4,6-dideoxy-α-D-mannopyranosyl-(1→2)-4-azido-3-O-benzyl-4,6-dideoxy-α-D-mannopyranosyl-(1→2)-4-azido-3-O-benzyl-4,6-dideoxy-α-D-mannopyranosyl-(1→2)-4-azido-3-O-benzyl-4,6-dideoxy-α-D-mannopyranosyl-(1→2)-4-azido-3-O-benzyl-4,6-dideoxy-α-D-mannopyranoside (**18**) 17

Trifluoroacetamidopropyl 4-azido-3-O-benzyl-4,6-dideoxy-α-D-mannopyranosyl-(1→2)-4-azido-3-O-benzyl-4,6-dideoxy-α-D-mannopyranosyl-(1→2)-4-azido-3-O-benzyl-4,6-dideoxy-α-D-mannopyranosyl-(1→2)-4-azido-3-O-benzyl-4,6-dideoxy-α-D-mannopyranosyl-(1→2)-4-azido-3-O-benzyl-4,6-dideoxy-α-D-mannopyranoside (**19**) 18

Trifluoroacetamidopropyl 4-amino-3-O-benzyl-4,6-dideoxy-α-D-mannopyranosyl-(1→2)-4-amino-3-O-benzyl-4,6-dideoxy-α-D-mannopyranoside (**20**) 19

3-Aminopropyl 4,6-dideoxy-4-formamido-α-D-mannopyranosyl-(1→2)-4,6-dideoxy-4-formamido-α-D-mannopyranoside (1a)

^{1}H NMR

D$_2$O, 600 MHz

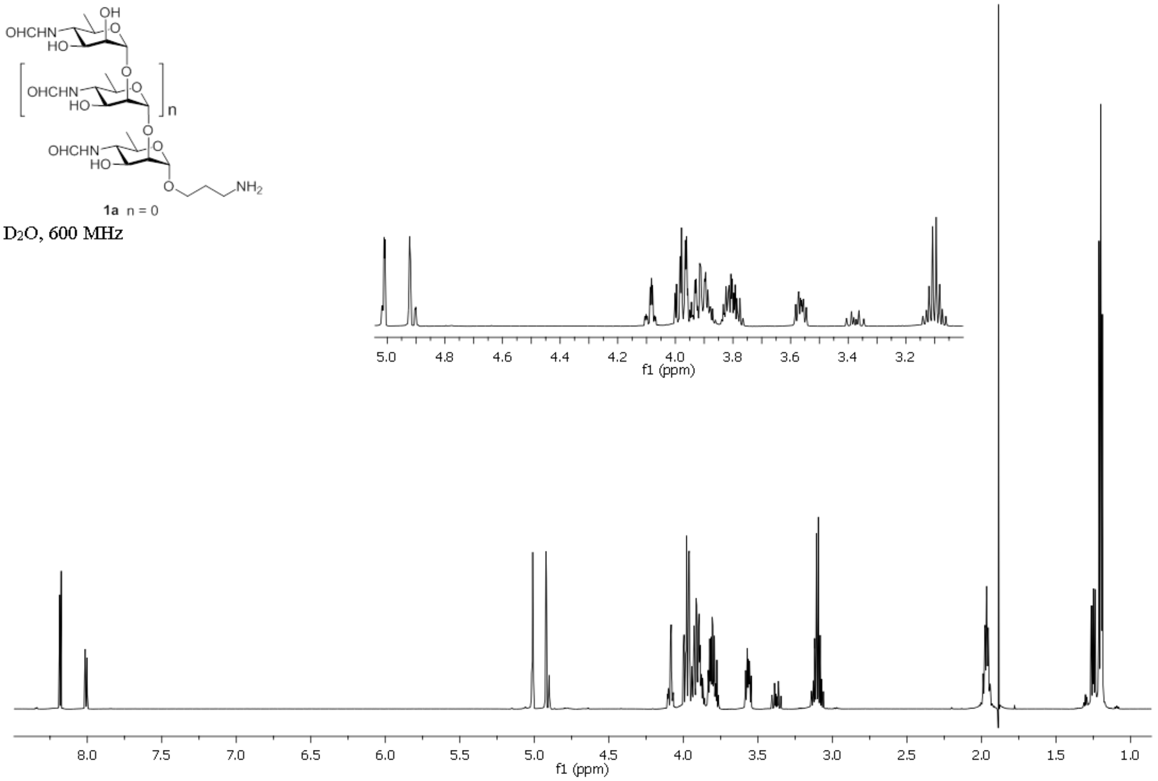

^{13}C NMR

D$_2$O, 150.9 MHz

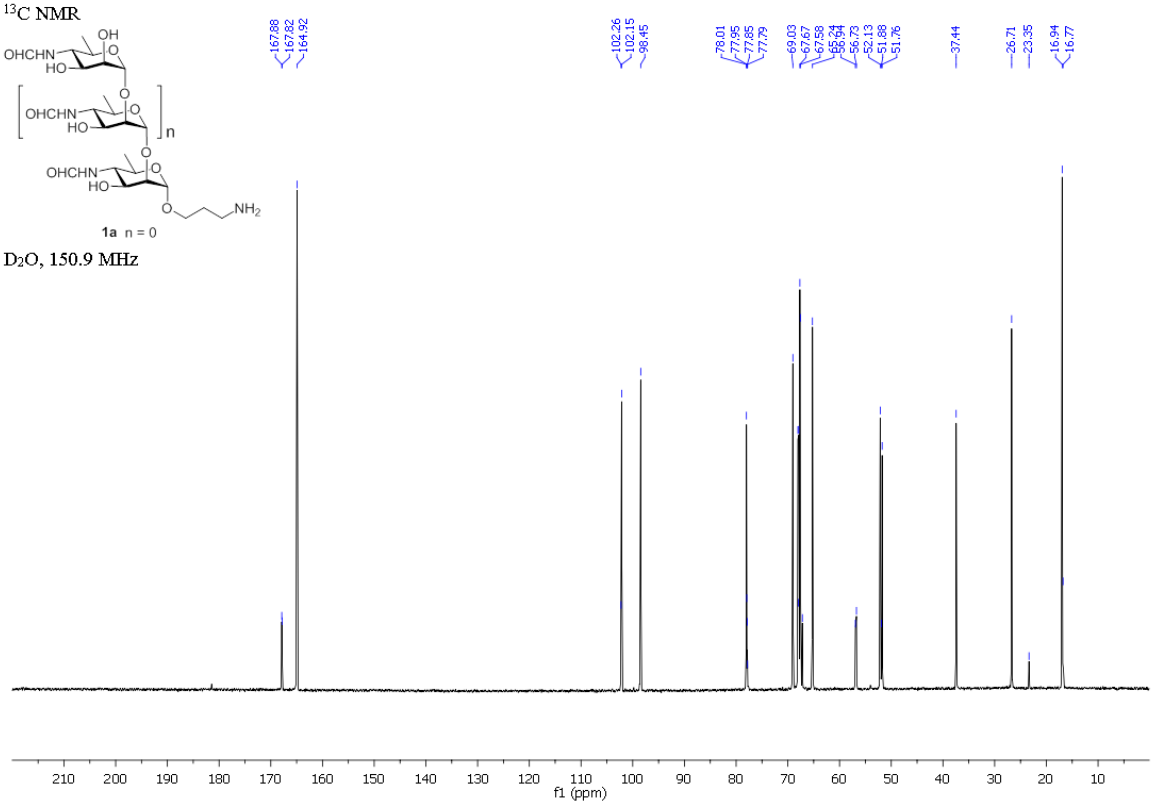

3-Aminopropyl 4,6-dideoxy-4-formamido-α-D-mannopyranosyl-(1→2)-4,6-dideoxy-4-formamido-α-D-mannopyranosyl-(1→2)-4,6-dideoxy-4-formamido-α-D-mannopyranoside (2a)

^{1}H NMR

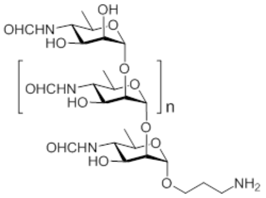

2a n = 1

D_2O, 600 MHz

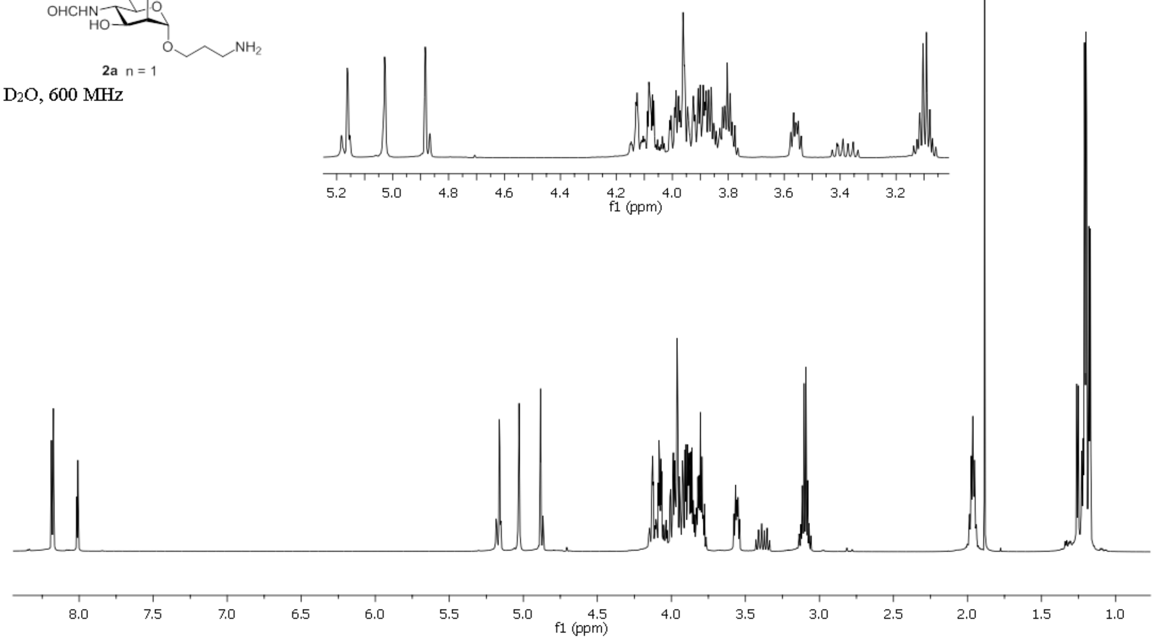

^{13}C NMR

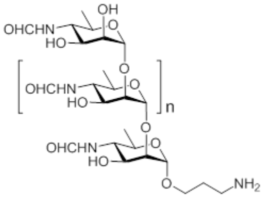

2a n = 1

D_2O, 150.9 MHz

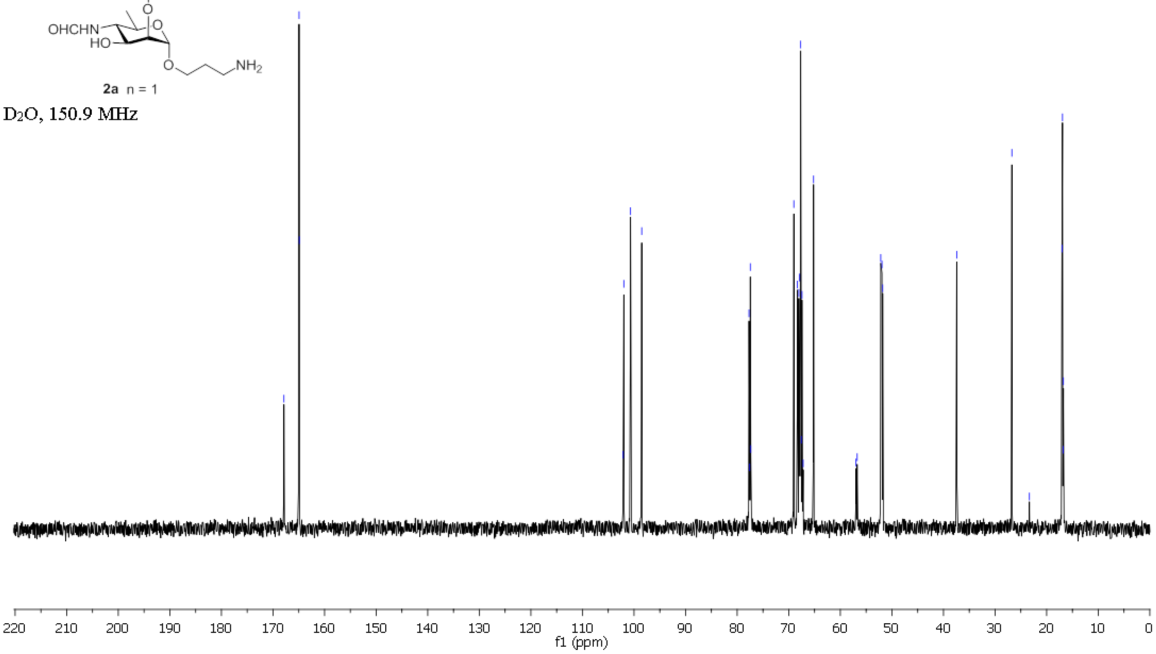

3-Aminopropyl 4,6-dideoxy-4-formamido-α-D-mannopyranosyl-(1→2)-4,6-dideoxy-4-formamido-α-D-mannopyranosyl-(1→2)-4,6-dideoxy-4-formamido-α-D-mannopyranosyl-(1→2)-4,6-dideoxy-4-formamido-α-D-mannopyranoside (3a)

^{13}C NMR

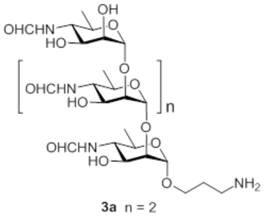

D$_2$O, 150.9 MHz

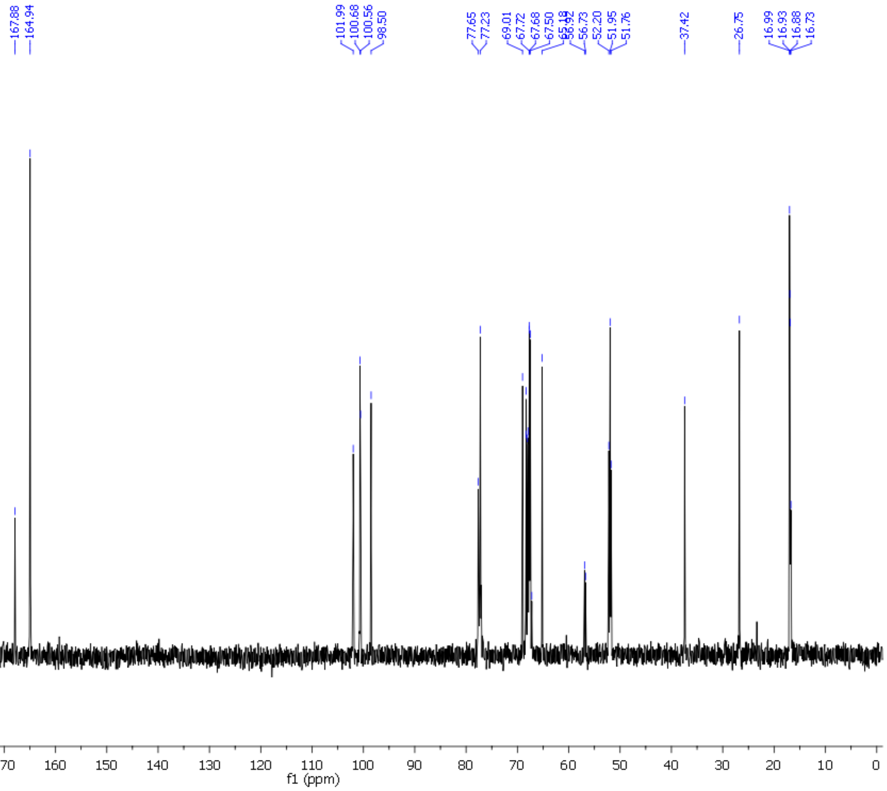

^{1}H NMR

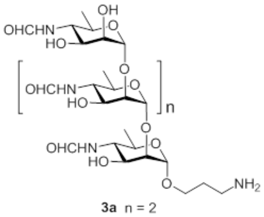

D$_2$O, 600 MHz

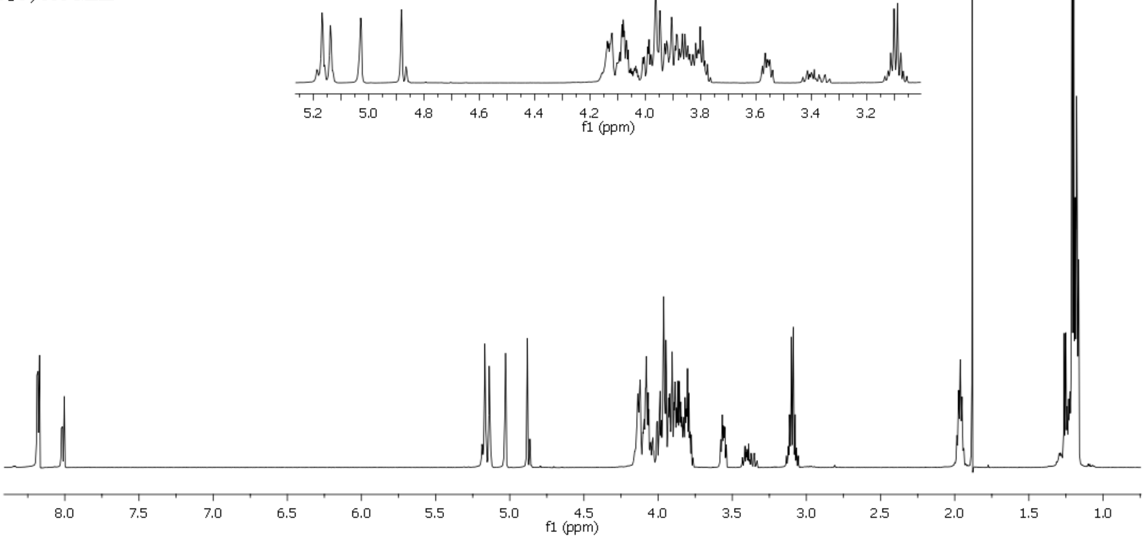

3-Aminopropyl 4,6-dideoxy-4-formamido-α-D-mannopyranosyl-(1→2)-4,6-dideoxy-4-formamido-α-D-mannopyranosyl-(1→2)-4,6-dideoxy-4-formamido-α-D-mannopyranosyl-(1→2)-4,6-dideoxy-4-formamido-α-D-mannopyranosyl-(1→2)-4,6-dideoxy-4-formamido-α-D-mannopyranoside (4a)

^{1}H NMR

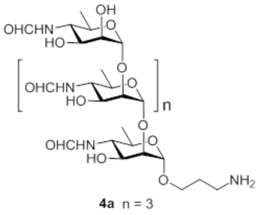

D$_2$O, 600 MHz

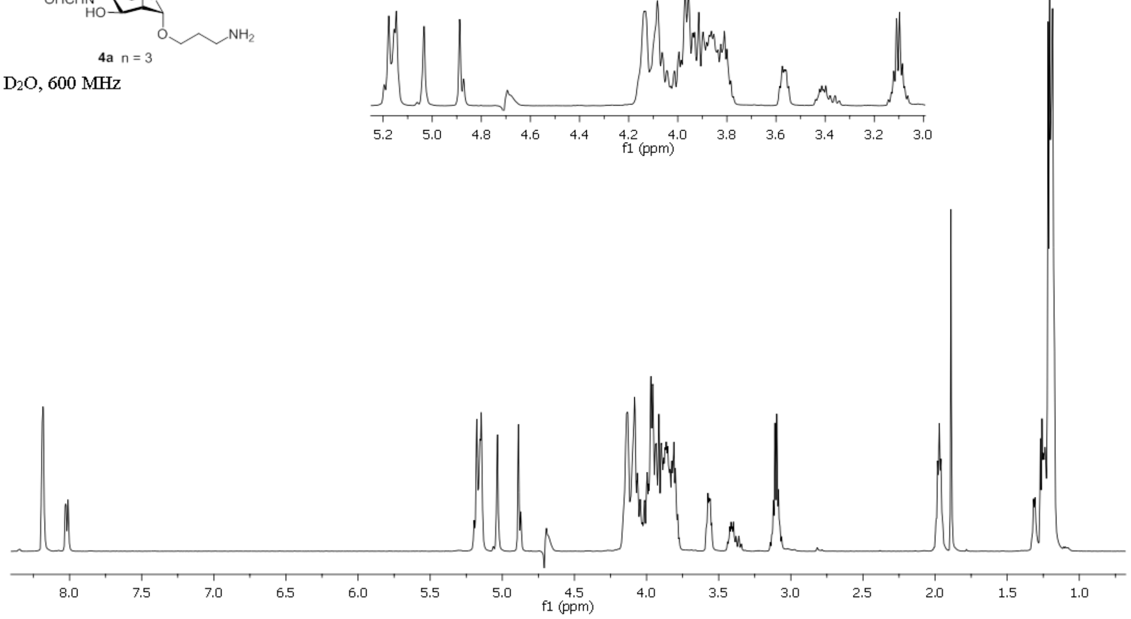

^{13}C NMR

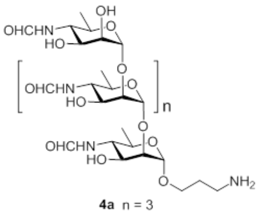

D$_2$O, 150.9 MHz

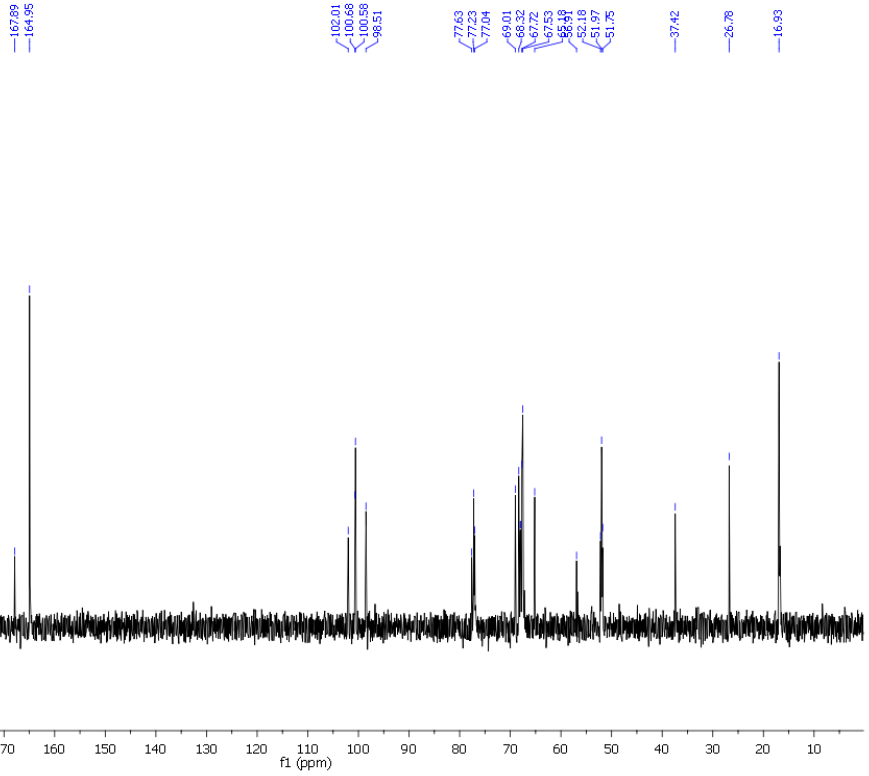

5-*tert*-Butyl-2-methylphenyl 2-O-acetyl-4-azido-3-O-benzyl-4,6-dideoxy-1-thio-α-D-mannopyranoside (6)

^{1}H NMR

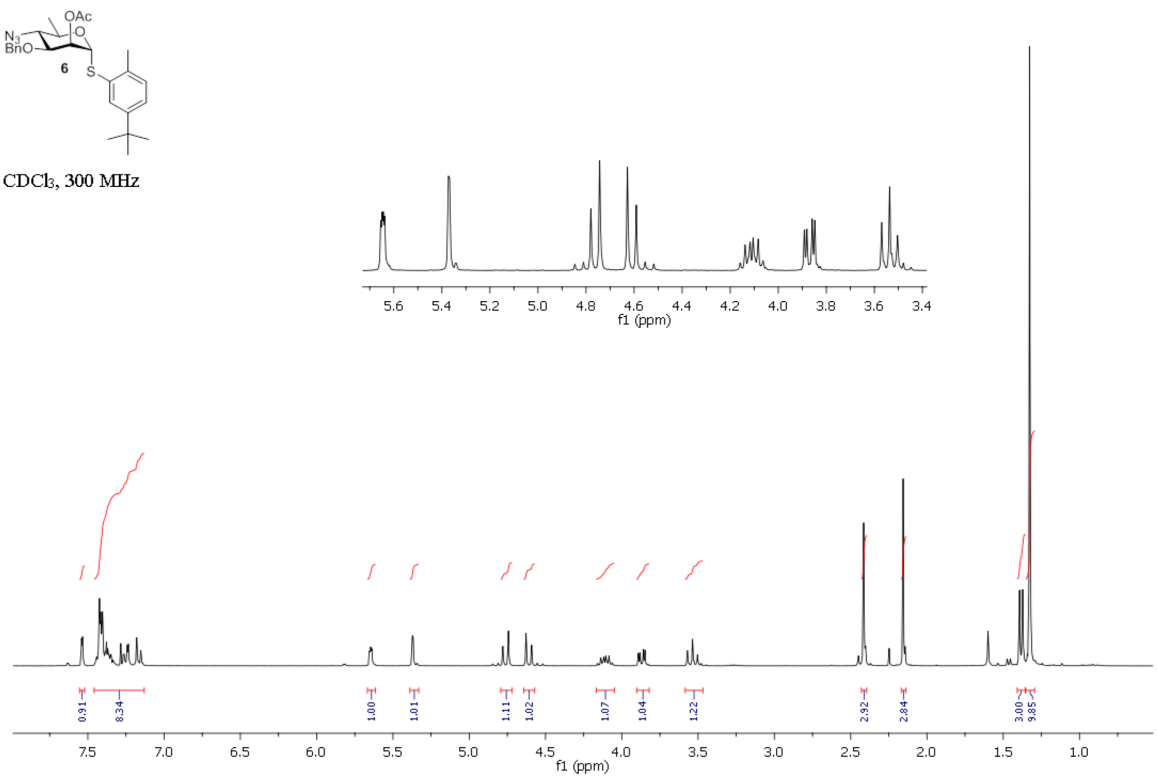

^{13}C NMR

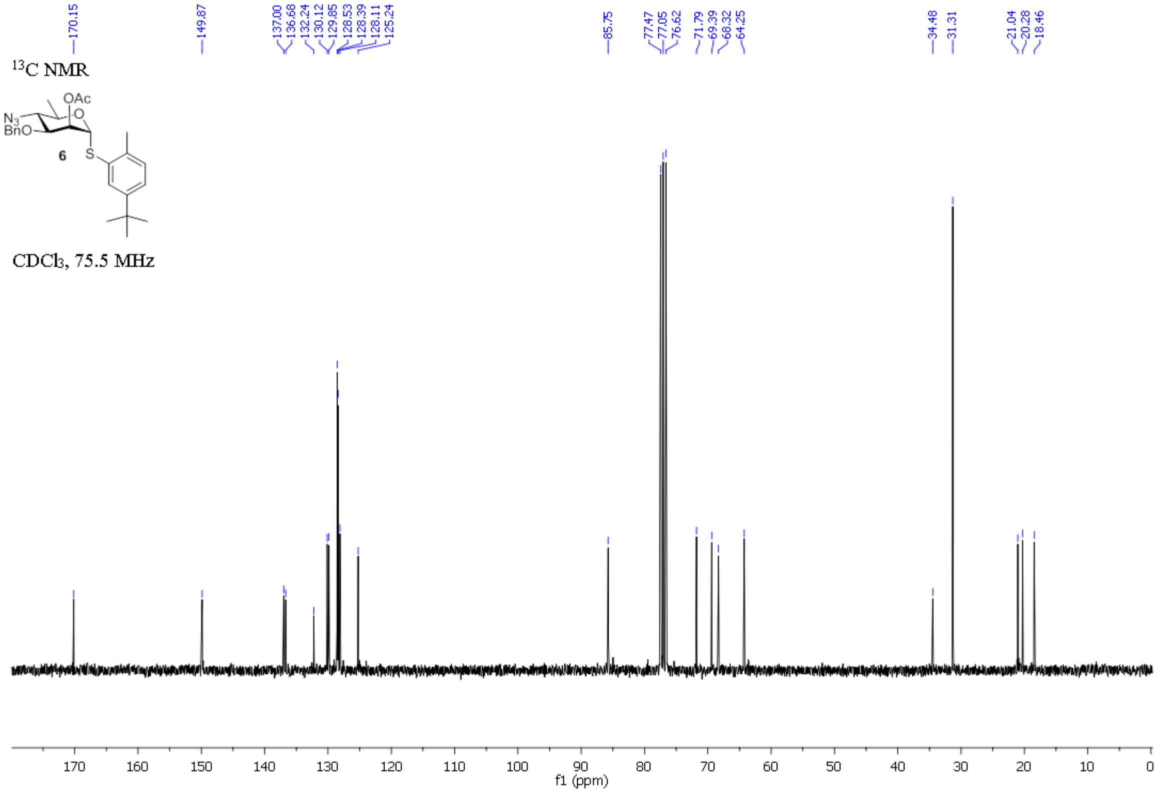

5-*tert*-Butyl-2-methylphenyl 4-azido-3-O-benzyl-4,6-dideoxy-1-thio-α-D-mannopyranoside (7)

^{1}H NMR

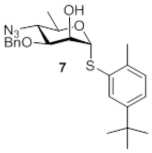

$CDCl_3$, 300 MHz

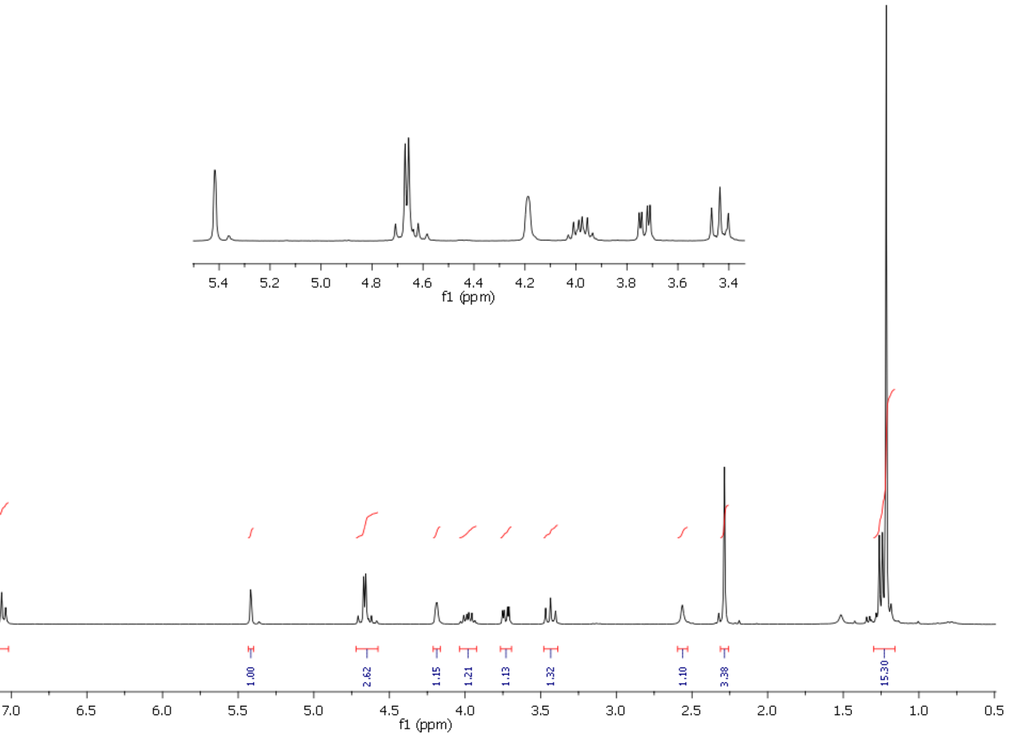

^{13}C NMR

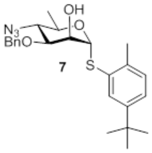

$CDCl_3$, 75.5 MHz

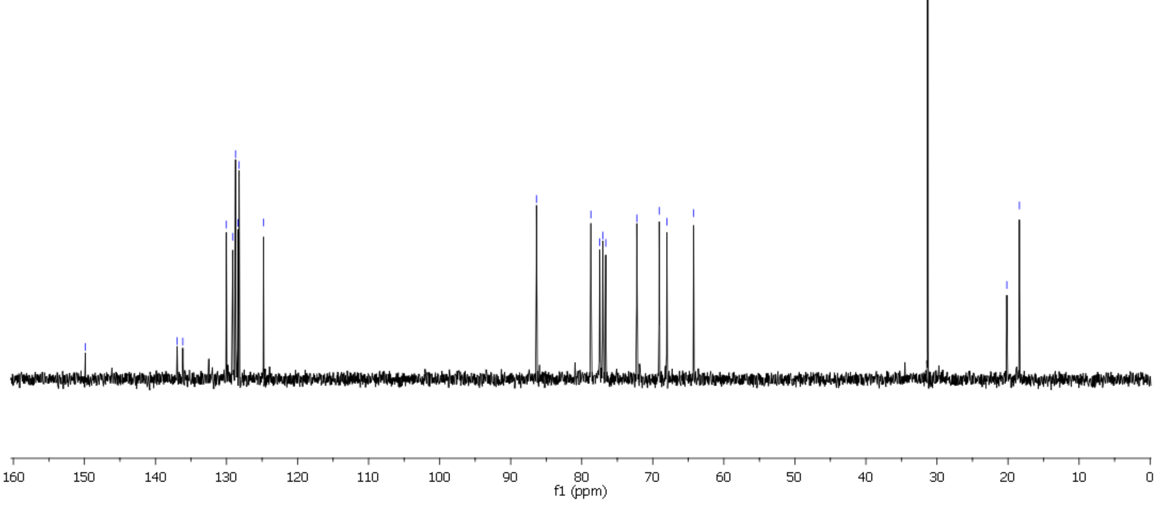

5-*tert*-Butyl-2-methylphenyl 2-O-acetyl-4-azido-3-O-benzyl-4,6-dideoxy-α-D-mannopyranosyl-(1→2)-4-azido-3-O-benzyl-4,6-dideoxy-1-thio-α-D-mannopyranoside (9)

^{1}H NMR

CDCl$_3$, 300 MHz

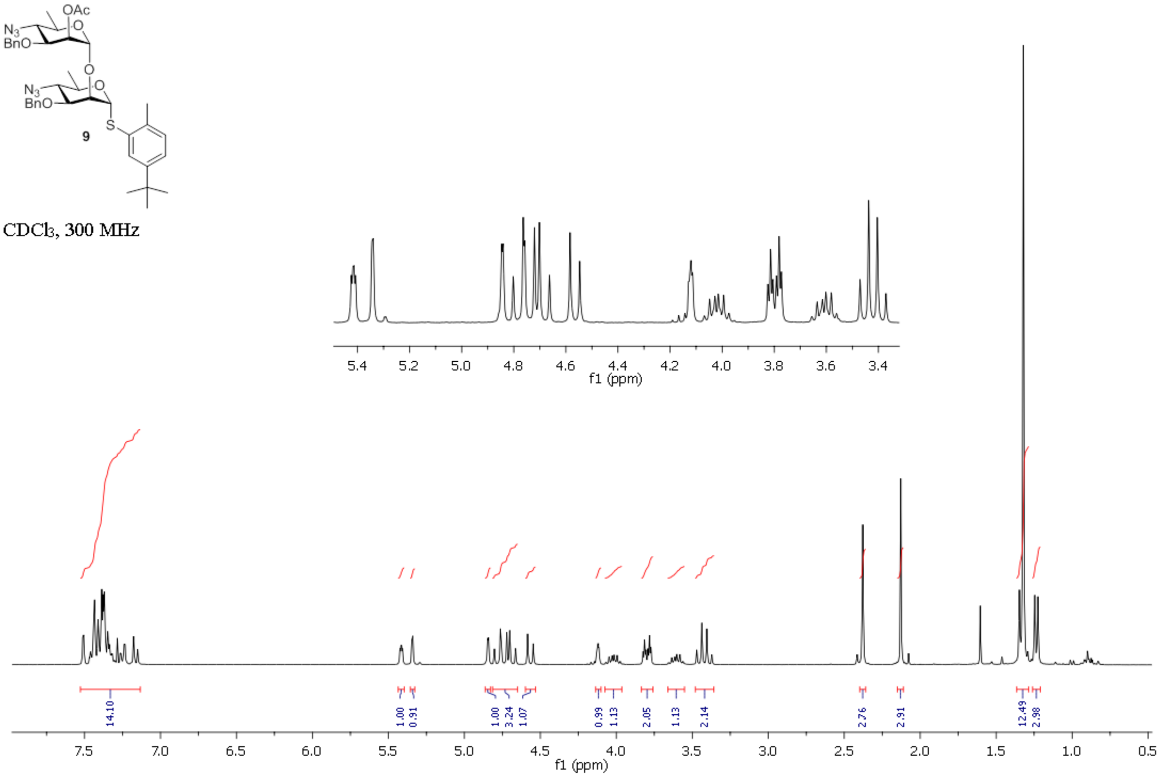

^{13}C NMR

CDCl$_3$, 75.5 MHz

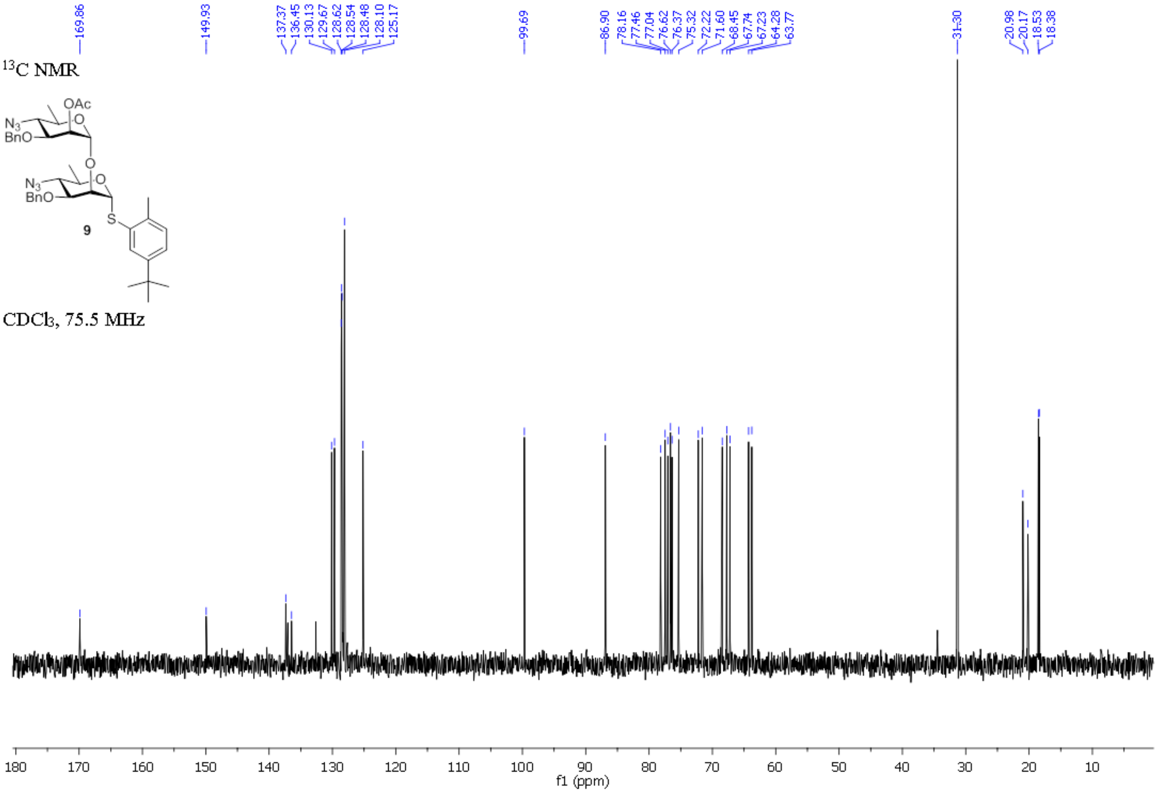

3-Trifluoroacetamidopropyl 2-O-acetyl-4-azido-3-O-benzyl-4,6-dideoxy-α-D-mannopyranosyl-(1→2)-4-azido-3-O-benzyl-4,6-dideoxy-α-D-mannopyranoside (11α)

^{1}H NMR

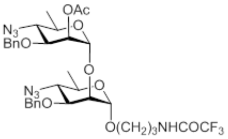

11α

CDCl$_3$, 300 MHz

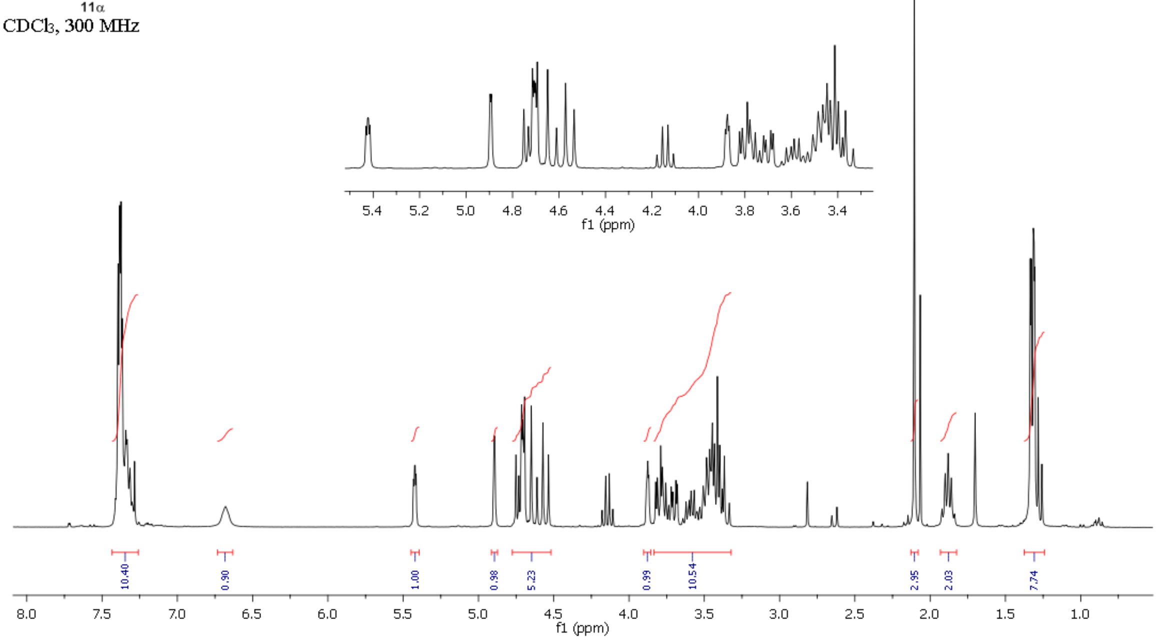

^{13}C NMR

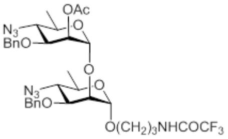

11α

CDCl$_3$, 75.5 MHz

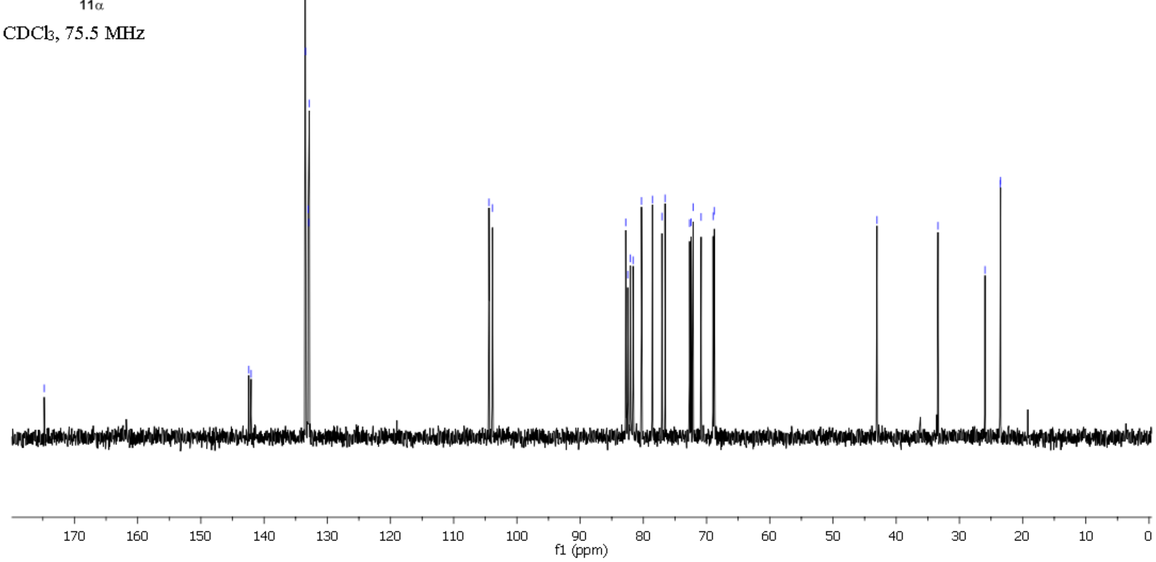

3-Trifluoroacetamidopropyl 2-O-acetyl-4-azido-3-O-benzyl-4,6-dideoxy-α-D-mannopyranosyl-(1→2)-4-azido-3-O-benzyl-4,6-dideoxy-β-D-mannopyranoside (11β)

^{1}H NMR

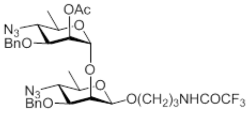

11β

$CDCl_3$, 300 MHz

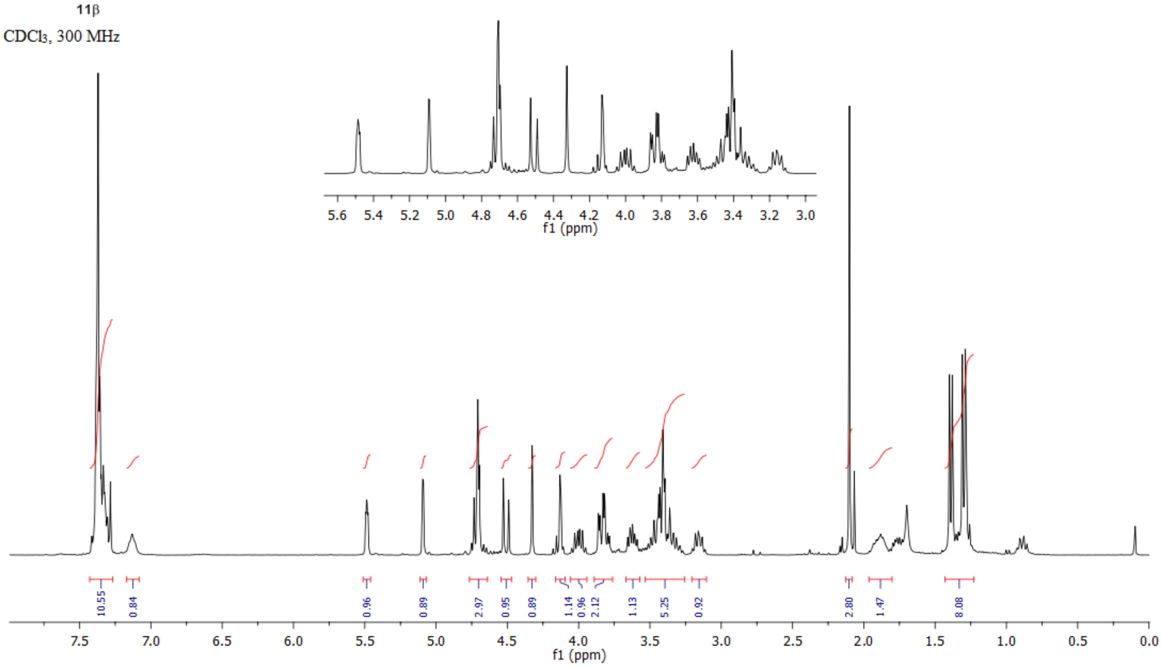

^{13}C NMR

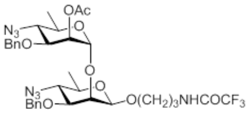

11β

$CDCl_3$, 75.5 MHz

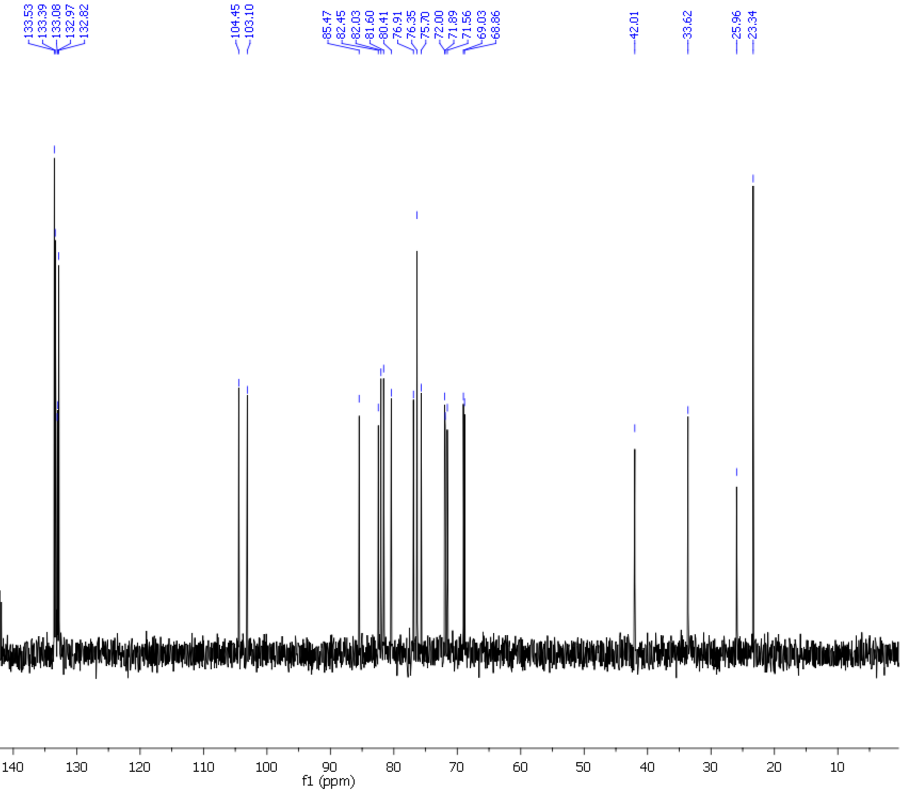

3-Trifluoroacetamidopropyl 4-azido-3-O-benzyl-4,6-dideoxy-α-D-mannopyranosyl-(1→2)-4-azido-3-O-benzyl-4,6-dideoxy-α-D-mannopyranoside (12)

^{1}H NMR

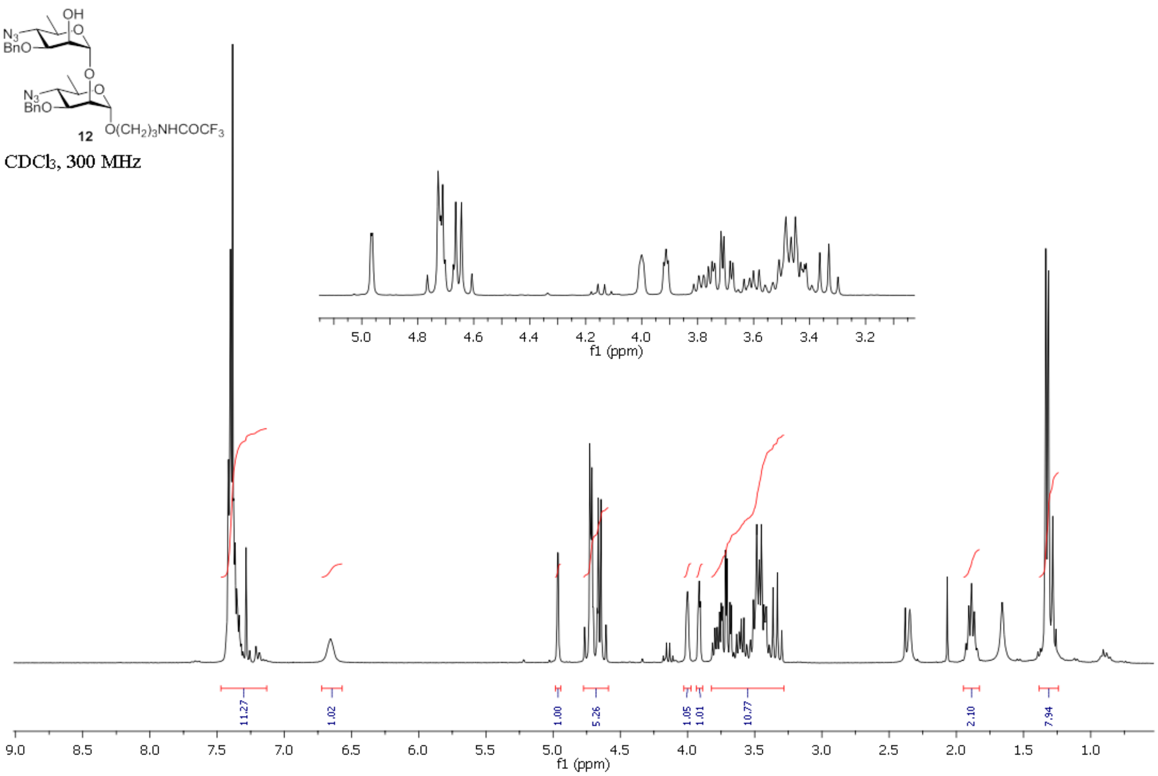

^{13}C NMR

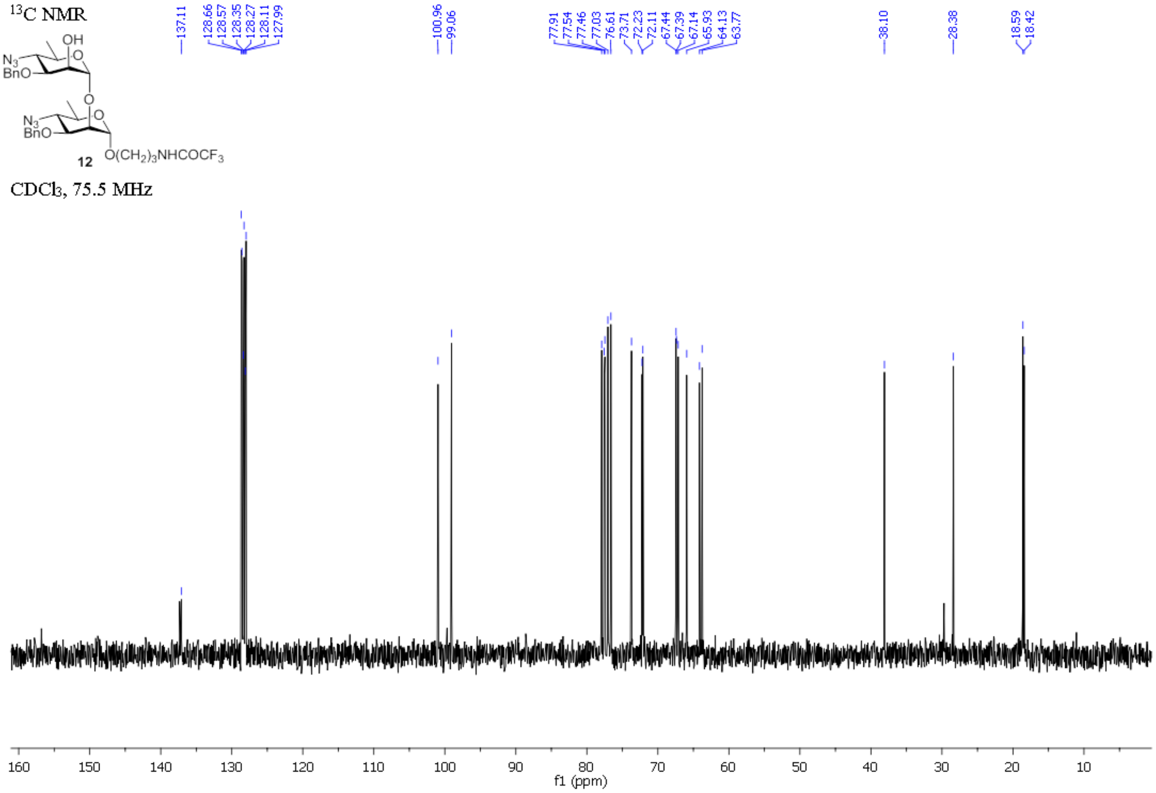

3-Trifluoroacetamidopropyl 2-O-acetyl-4-azido-3-O-benzyl-4,6-dideoxy-α-D-mannopyranosyl-(1→2)-4-azido-3-O-benzyl-4,6-dideoxy-α-D-mannopyranosyl-(1→2)-4-azido-3-O-benzyl-4,6-dideoxy-α-D-mannopyranoside (14)

^{1}H NMR

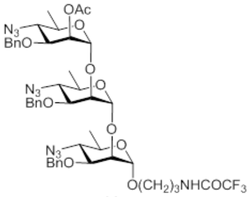

14

$CDCl_3$, 600 MHz

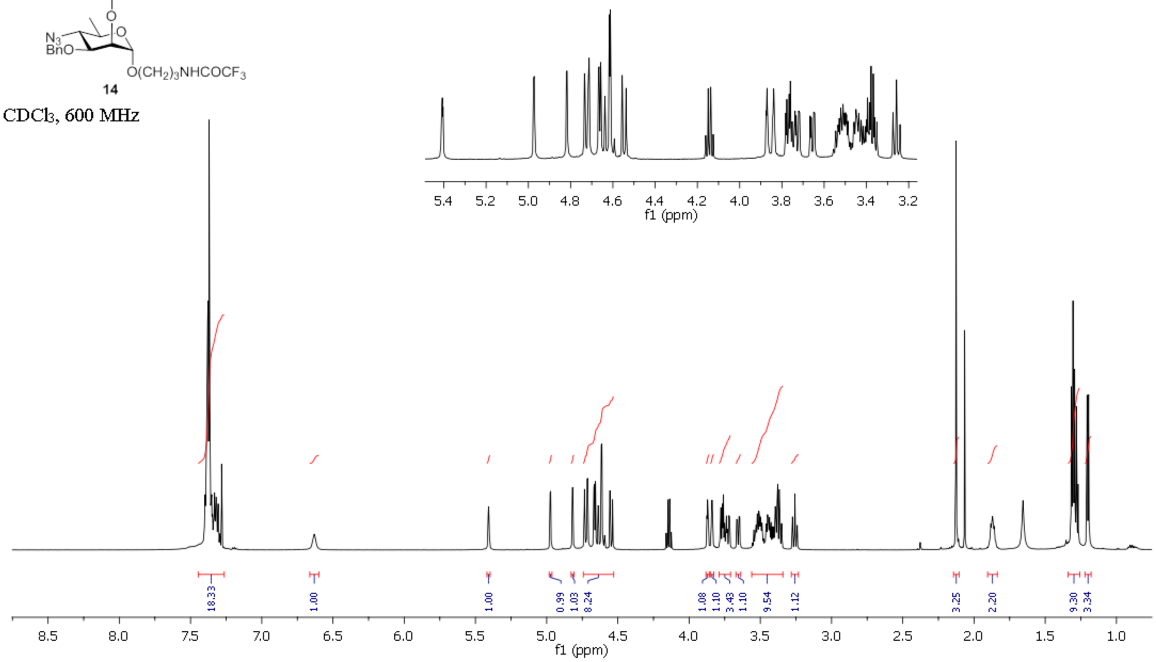

^{13}C NMR

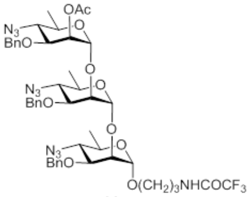

14

$CDCl_3$, 150.9 MHz

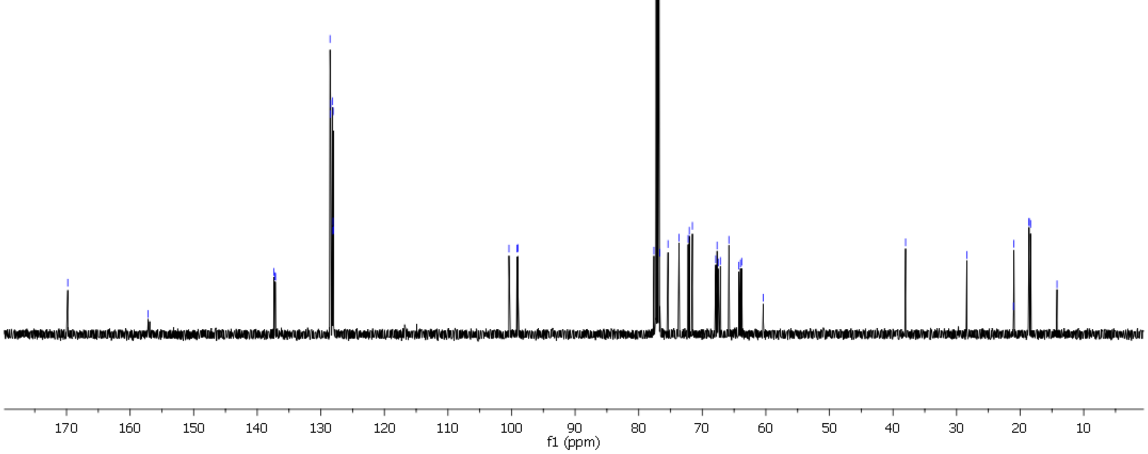

3-Trifluoroacetamidopropyl 4-azido-3-O-benzyl-4,6-dideoxy-α-D-mannopyranosyl-(1→2)-4-azido-3-O-benzyl-4,6-dideoxy-α-D-mannopyranosyl-(1→2)-4-azido-3-O-benzyl-4,6-dideoxy-α-D-mannopyranoside (15)

^{1}H NMR

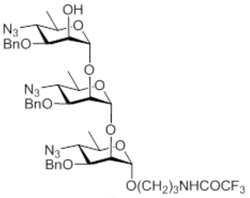

15

CDCl$_3$, 400 MHz

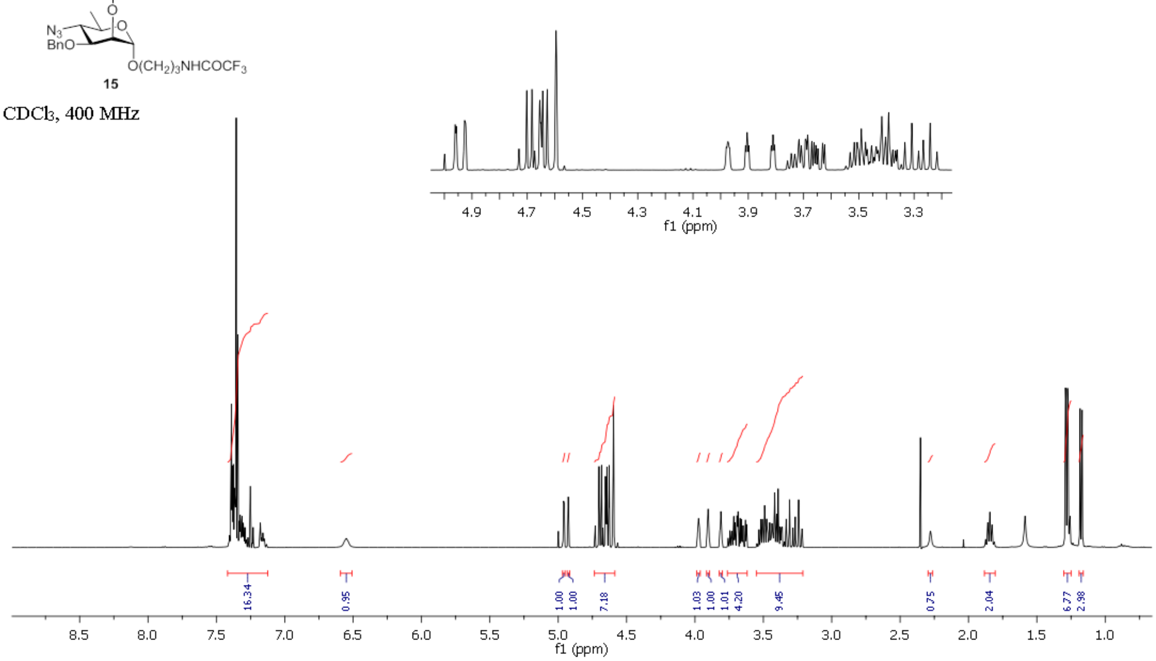

^{13}C NMR

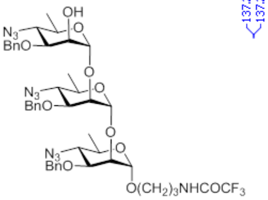

15

CDCl$_3$, 100.6 MHz

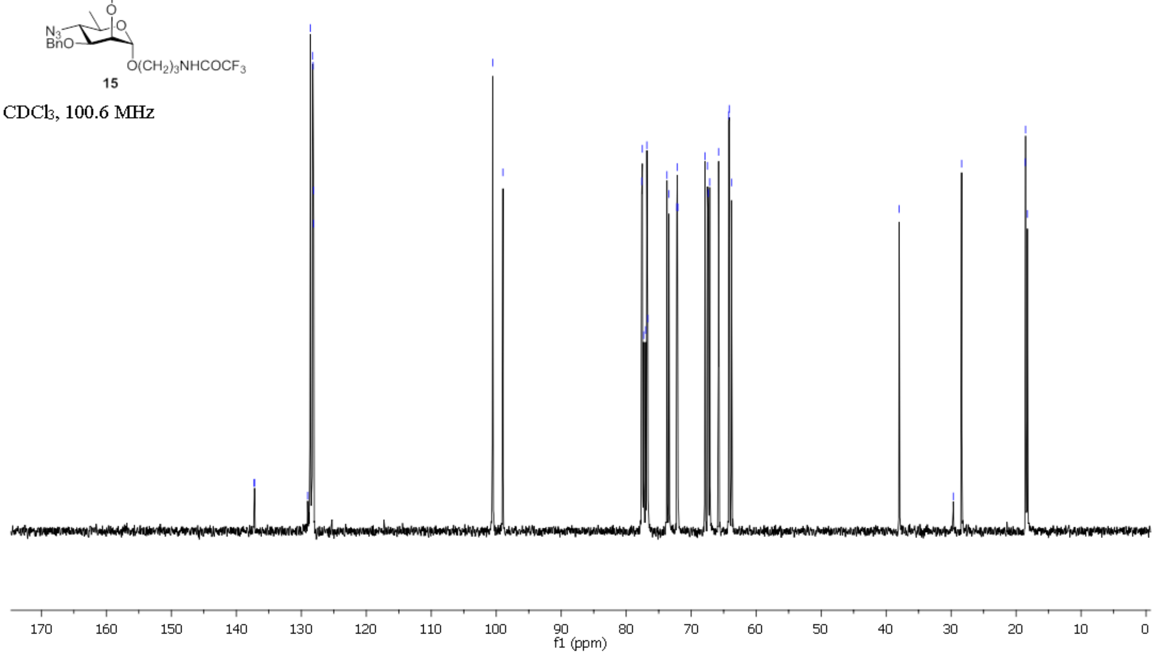

3-Trifluoroacetamidopropyl 2-O-acetyl-4-azido-3-O-benzyl-4,6-dideoxy-α-D-mannopyranosyl-(1→2)-4-azido-3-O-benzyl-4,6-dideoxy-α-D-mannopyranosyl-(1→2)-4-azido-3-O-benzyl-4,6-dideoxy-α-D-mannopyranosyl-(1→2)-4-azido-3-O-benzyl-4,6-dideoxy-α-D-mannopyranoside (16)

^{1}H NMR

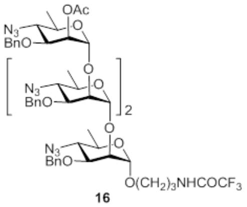

16

$CDCl_3$, 600 MHz

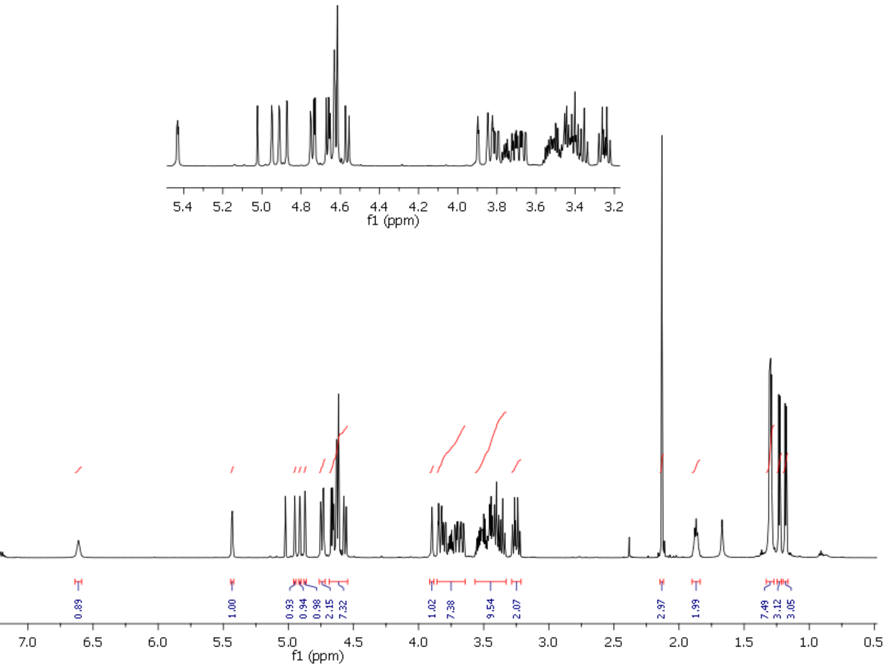

^{13}C NMR

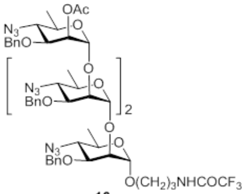

16

$CDCl_3$, 150.9 MHz

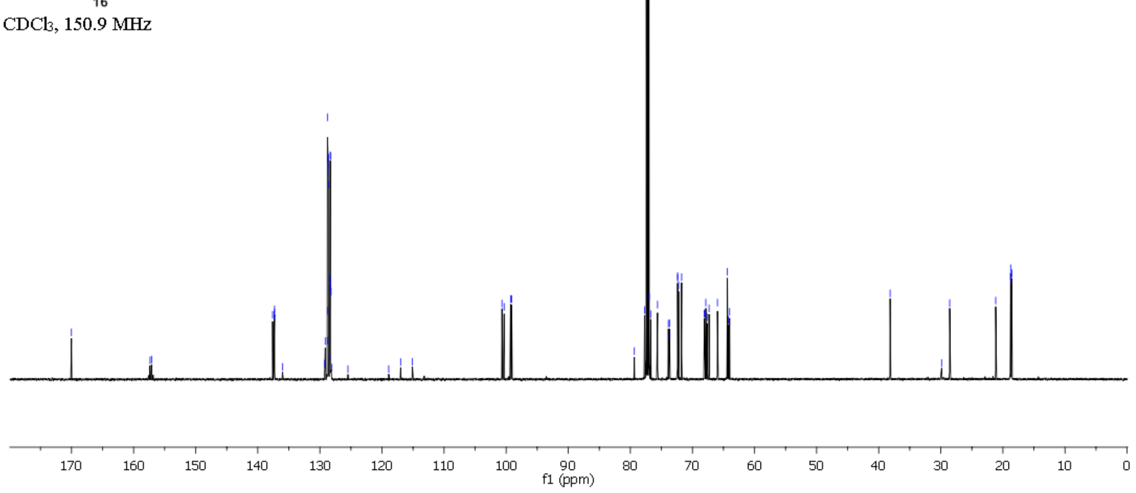

3-Trifluoroacetamidopropyl 4-azido-3-O-benzyl-4,6-dideoxy-α-D-mannopyranosyl-(1→2)-4-azido-3-O-benzyl-4,6-dideoxy-α-D-mannopyranosyl-(1→2)-4-azido-3-O-benzyl-4,6-dideoxy-α-D-mannopyranosyl-(1→2)-4-azido-3-O-benzyl-4,6-dideoxy-α-D-mannopyranoside (17)

^{1}H NMR

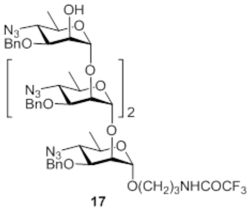

17

CDCl$_3$, 400 MHz

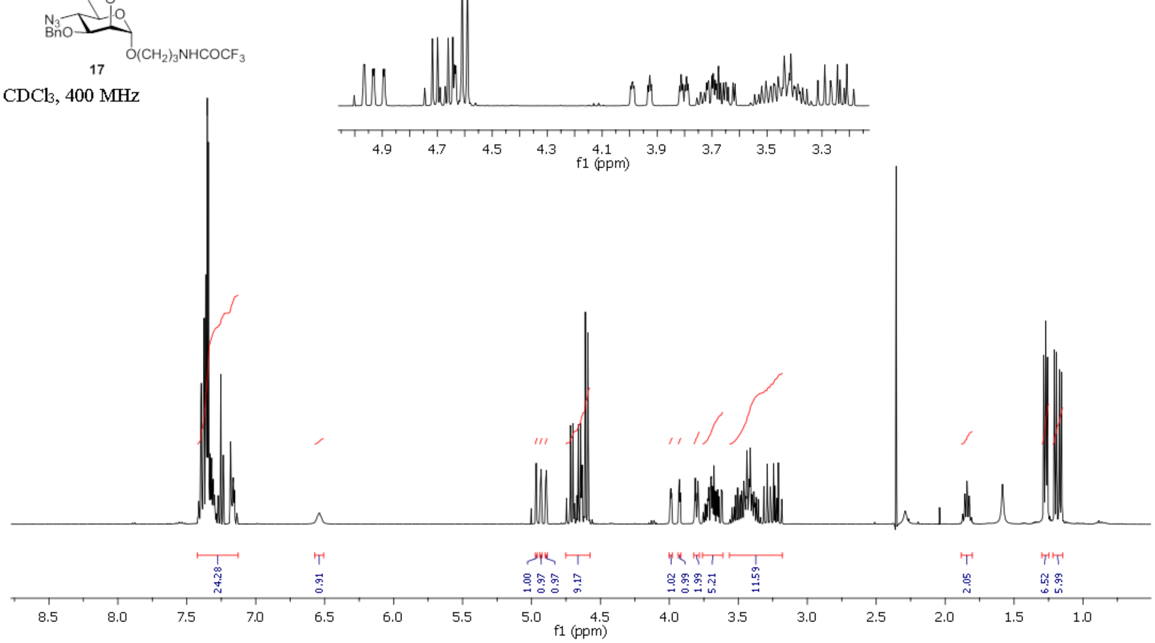

^{13}C NMR

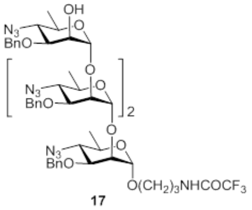

17

CDCl$_3$, 100.6 MHz

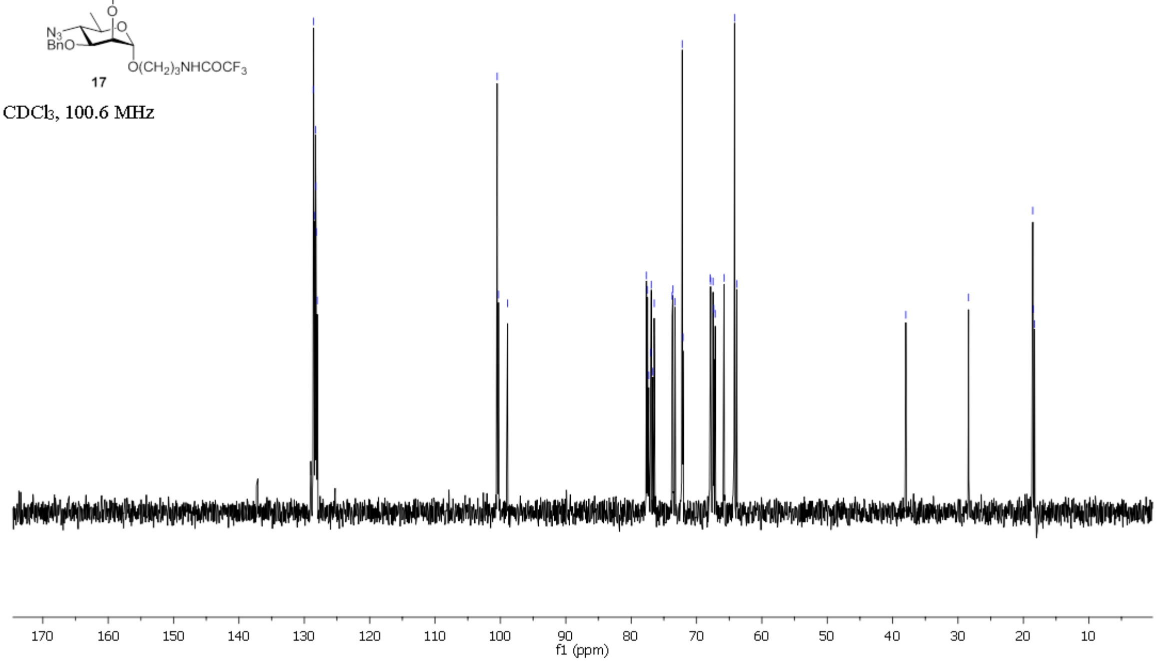

3-Trifluoroacetamidopropyl 2-O-acetyl-4-azido-3-O-benzyl-4,6-dideoxy-α-D-mannopyranosyl-(1→2)-4-azido-3-O-benzyl-4,6-dideoxy-α-D-mannopyranosyl-(1→2)-4-azido-3-O-benzyl-4,6-dideoxy-α-D-mannopyranosyl-(1→2)-4-azido-3-O-benzyl-4,6-dideoxy-α-D-mannopyranosyl-(1→2)-4-azido-3-O-benzyl-4,6-dideoxy-α-D-mannopyranoside (18)

^{1}H NMR

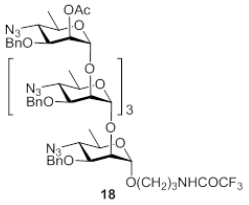

CDCl$_3$, 400 MHz

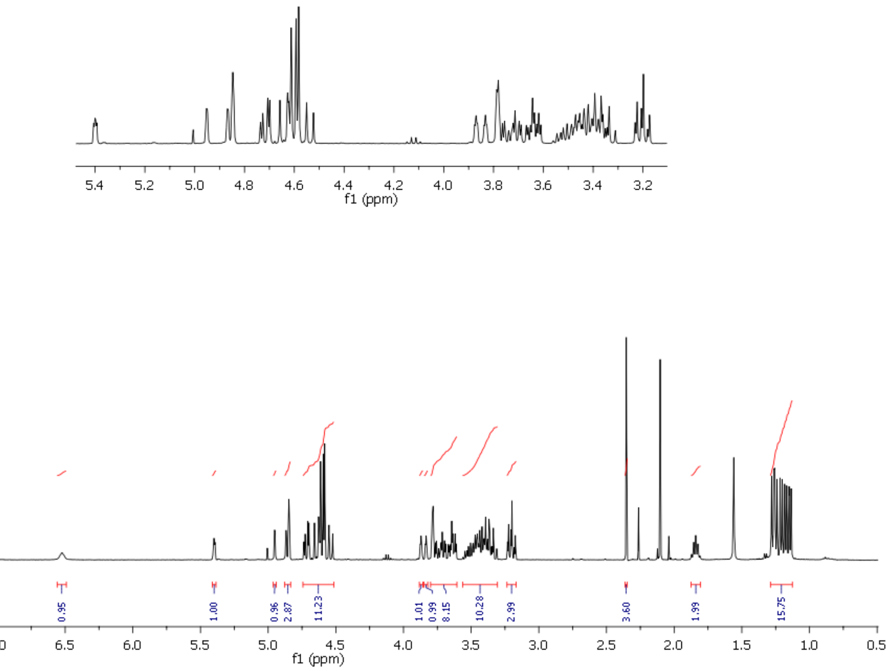

^{13}C NMR

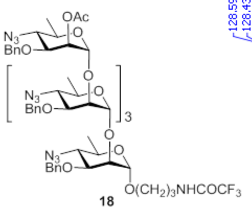

CDCl$_3$, 100.6 MHz

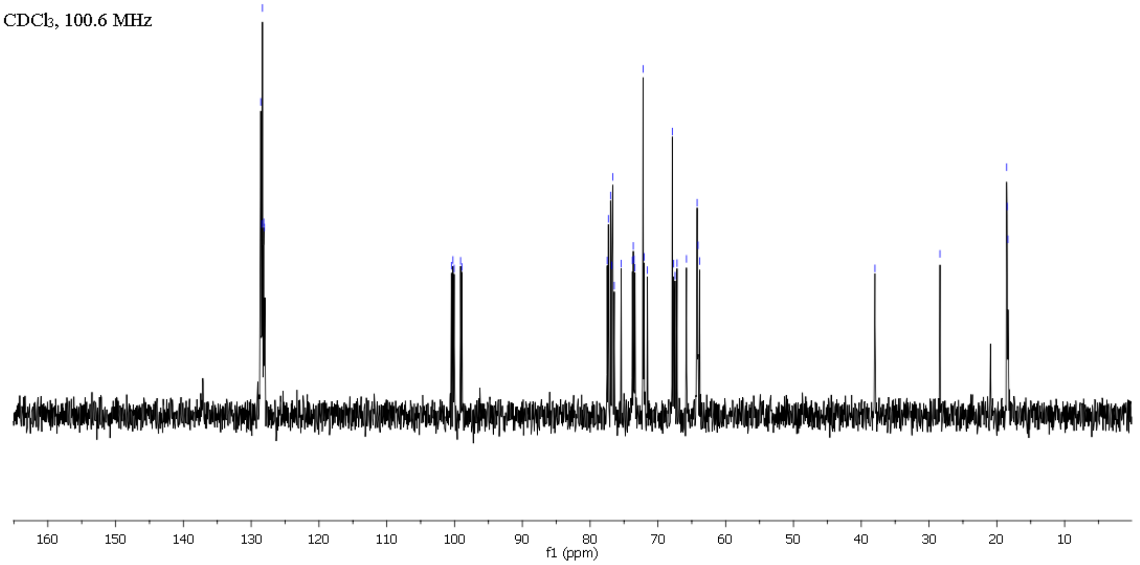

3-Trifluoroacetamidopropyl 4-azido-3-O-benzyl-4,6-dideoxy-α-D-mannopyranosyl-(1→2)-4-azido-3-O-benzyl-4,6-dideoxy-α-D-mannopyranosyl-(1→2)-4-azido-3-O-benzyl-4,6-dideoxy-α-D-mannopyranosyl-(1→2)-4-azido-3-O-benzyl-4,6-dideoxy-α-D-mannopyranosyl-(1→2)-4-azido-3-O-benzyl-4,6-dideoxy-α-D-mannopyranoside (19)

^{1}H NMR

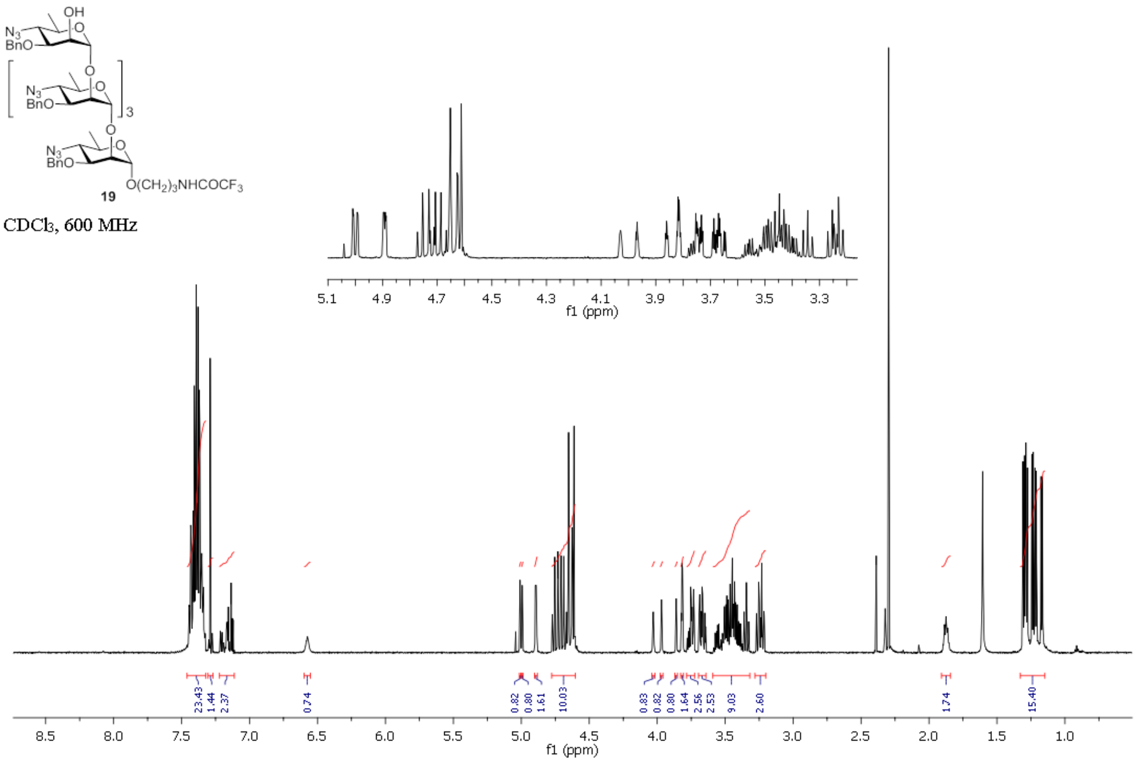

^{13}C NMR

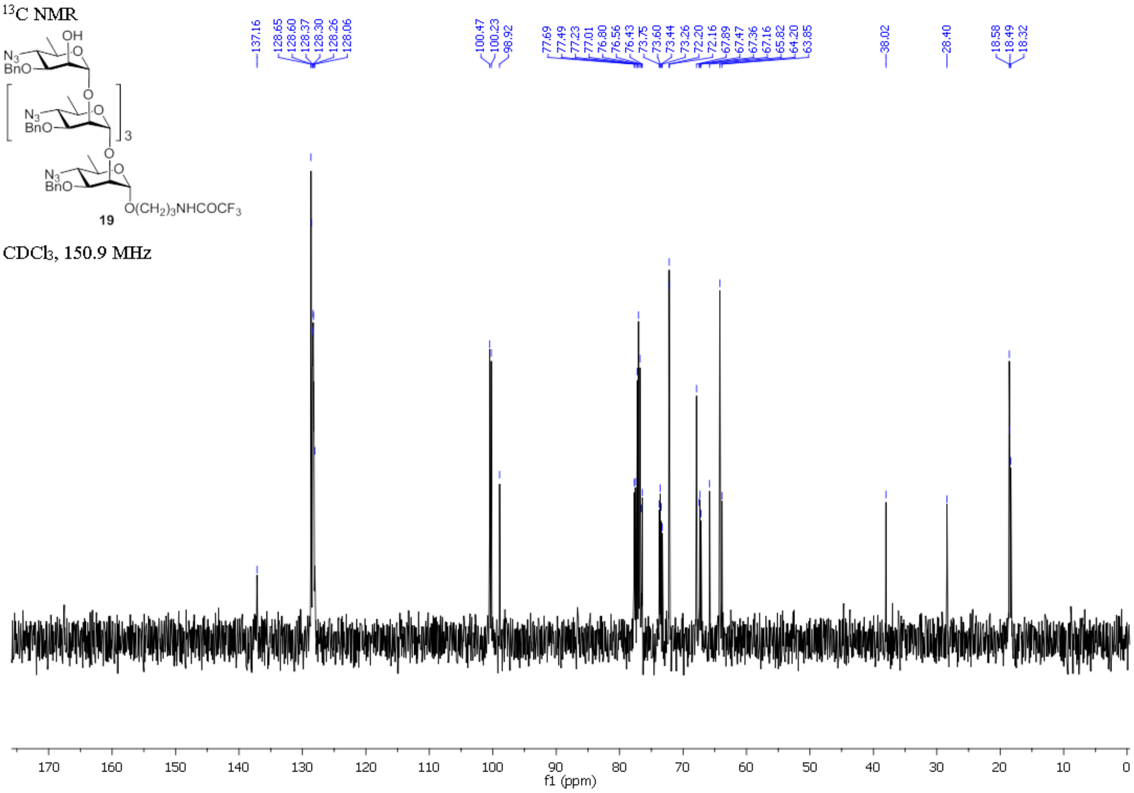

3-Trifluoroacetamidopropyl 4-amino-3-O-benzyl-4,6-dideoxy-α-D-mannopyranosyl-(1→2)-4-amino-3-O-benzyl-4,6-dideoxy-α-D-mannopyranoside (20)

^{1}H NMR

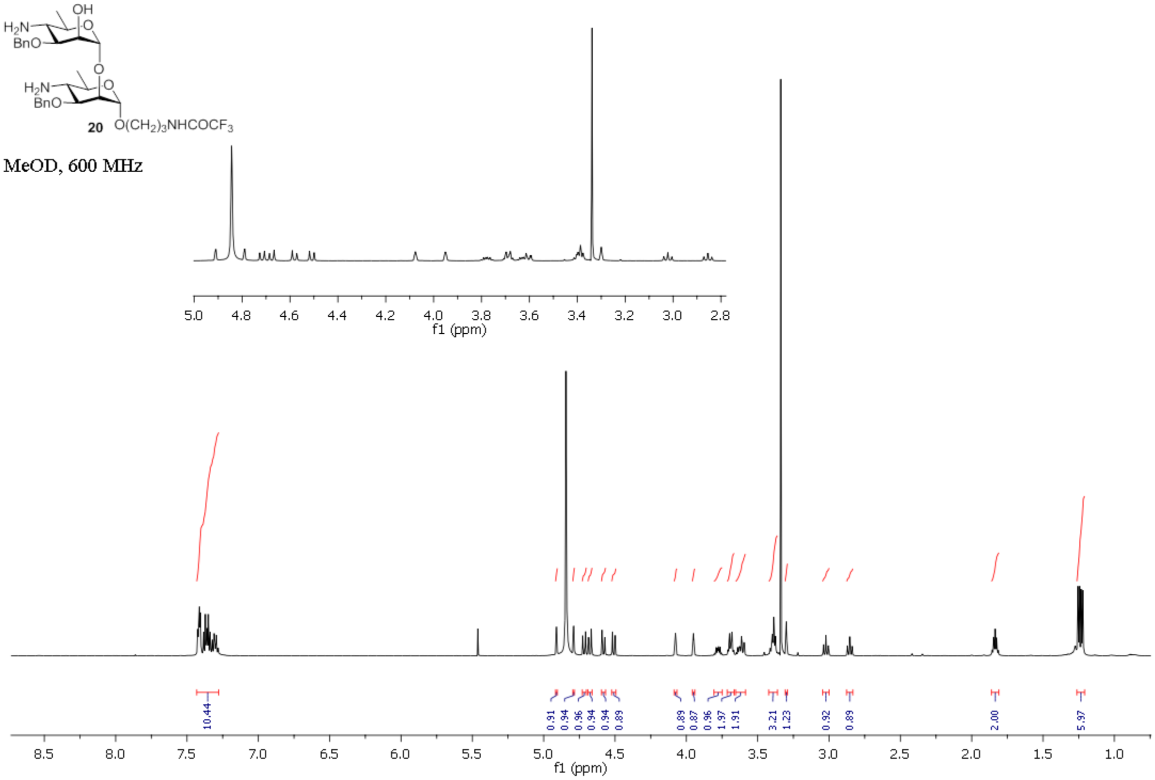

^{13}C NMR

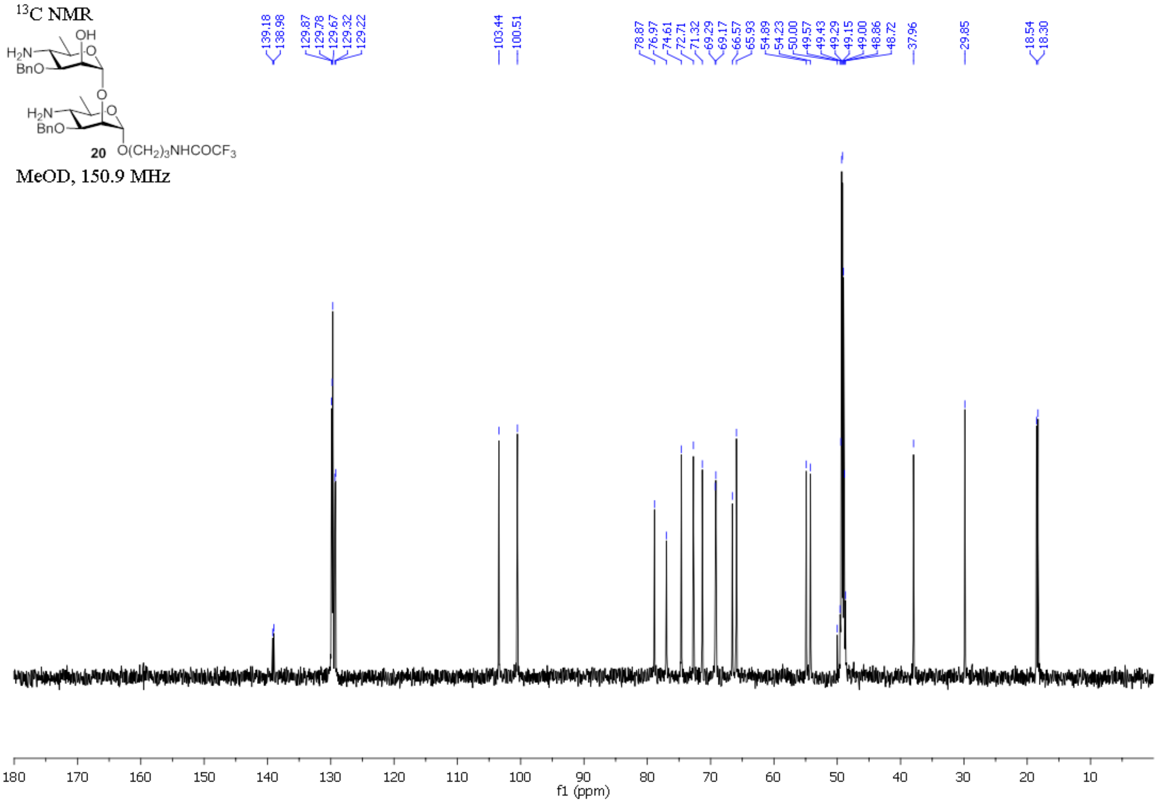

Supplement: Supplementary file 1 [file DataSheet1.pdf]
